# Flux-Tunable Josephson Diode Effect in a Hybrid Four-Terminal Josephson Junction

**DOI:** 10.1021/acsnano.4c01642

**Published:** 2024-03-15

**Authors:** Marco Coraiola, Aleksandr E. Svetogorov, Daniel Z. Haxell, Deividas Sabonis, Manuel Hinderling, Sofieke C. ten Kate, Erik Cheah, Filip Krizek, Rüdiger Schott, Werner Wegscheider, Juan Carlos Cuevas, Wolfgang Belzig, Fabrizio Nichele

**Affiliations:** † IBM Research Europe—Zurich, 8803 Rüschlikon, Switzerland; ‡ Fachbereich Physik, Universität Konstanz, D-78457 Konstanz, Germany; ¶ Laboratory for Solid State Physics, ETH Zürich, 8093 Zürich, Switzerland; § Institute of Physics, Czech Academy of Sciences, 162 00 Prague, Czech Republic; ∥ Departamento de Física Teórica de la Materia Condensada and Condensed Matter Physics Center (IFIMAC), Universidad Autónoma de Madrid, E-28049 Madrid, Spain

**Keywords:** superconducting diode effect, multiterminal Josephson junction, superconductor−semiconductor hybrid, 2DEG, nonreciprocal transport

## Abstract

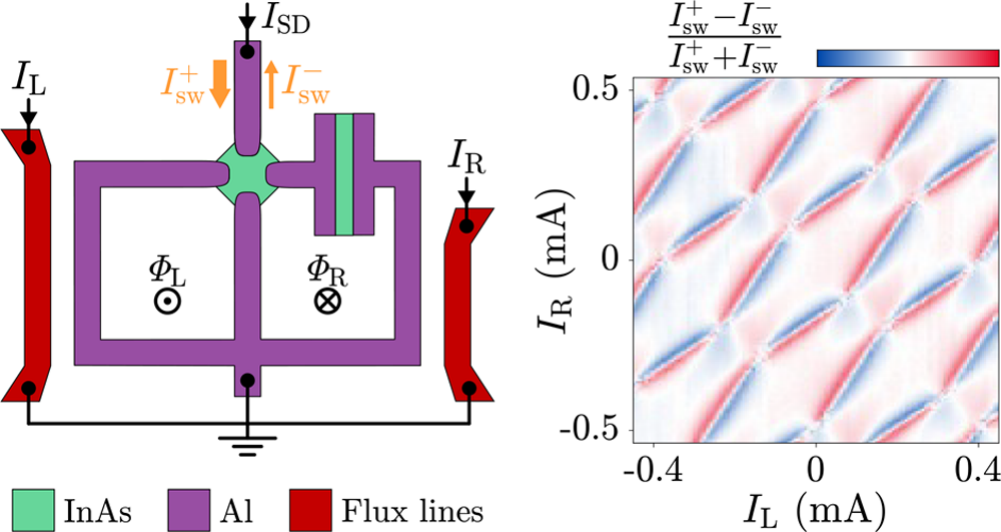

We investigate the direction-dependent switching current in a flux-tunable four-terminal Josephson junction defined in an InAs/Al two-dimensional heterostructure. The device exhibits the Josephson diode effect with switching currents that depend on the sign of the bias current. The superconducting diode efficiency, reaching a maximum of |η| ≈ 34%, is widely tunable—both in amplitude and sign—as a function of magnetic fluxes and gate voltages. Our observations are supported by a circuit model of three parallel Josephson junctions with nonsinusoidal current–phase relation. With respect to conventional Josephson interferometers, phase-tunable multiterminal Josephson junctions enable large diode efficiencies in structurally symmetric devices, where local magnetic fluxes generated on the chip break both time-reversal and spatial symmetries. Our work presents an approach for developing Josephson diodes with wide-range tunability that do not rely on exotic materials.

Nonreciprocal transport phenomena play a key role in modern electronics, with semiconductor diodes serving as the fundamental components for numerous devices.^1^ In analogy to the semiconductor diode, whose electrical resistance strongly depends on the current direction, a superconducting diode allows a larger supercurrent to flow in one direction compared to the other.^2^ Nonreciprocal supercurrents were recognized already in the 1970s in superconducting quantum interference devices (SQUIDs) based on superconducting bridges^3^ and tunnel Josephson junctions (JJs),^4,5^ arising as a consequence of the finite loop inductance. Direction-dependent switching currents were also observed in conventional superconducting thin films and interpreted as a manifestation of microscopic asymmetries in the device geometry.^6^ More recently, the superconducting diode effect (SDE) has sparked renewed interest, driven by its connection to the fundamental properties of a diverse range of superconducting systems, where the breaking of both inversion and time-reversal symmetries is required for the effect to occur. Since its observation in superconducting multilayers,^7^ the SDE has been the subject of thorough experimental and theoretical investigation, both in junction-free thin films^8−13^ and JJs based on semiconductors with spin–orbit coupling,^14−17^ finite-momentum superconductors,^18−20^ or multilayered materials, realizing sizable asymmetries even without external magnetic fields.^21−28^ An alternative platform proposed to achieve the SDE in Josephson devices—where it is usually referred to as the Josephson diode effect (JDE)—relies on a supercurrent interferometer, where two JJs with nonsinusoidal current–phase relations (CPRs) are combined in a SQUID.^29,30^ Such CPRs, containing contributions from higher harmonics than the conventional 2π-periodic component, are routinely attained in high-quality superconductor–semiconductor planar materials,^31−33^ where hybrid JJs host Andreev bound states (ABSs) characterized by high transmission. Key ingredients for the JDE to occur in this system are the different harmonic content between the two JJs and a magnetic flux threading the SQUID loop,^29^ as recently demonstrated in two-dimensional (2D) electron^34,35^ and hole^36^ systems, obtaining large diode efficiencies at equilibrium up to approximately 30%.

Multiterminal JJs are emerging as a promising platform to investigate supercurrent nonreciprocities. Initial experiments^37^ identified the JDE and ascribed it to phase-drag effects.^38^ Coupling between JJs hosting ABSs realizes Andreev molecules,^39−41^ which also give rise to the JDE.^42,43^ Another line of research focused on multiterminal devices featuring more than two current ports.^44,45^ In this configuration, a bias current applied to one lead controls the switching current and its nonreciprocity measured between two other leads. Furthermore, the JDE was achieved by engineering higher harmonics in the CPR of a three-terminal JJ network threaded by a magnetic flux.^46^

Significant potential of multiterminal JJs lies in the ability to manipulate multiple phase differences. This capability would enable the engineering of an all-flux-tunable Josephson diode, offering versatile device design and control. In this work, we fill this gap by realizing a superconductor–semiconductor four-terminal JJ (4TJJ) embedded in a double-loop geometry, where two superconducting phase differences are independently controlled via integrated flux-bias lines. Our device, operated in a two-terminal configuration (i.e., a single bias current is required), leverages the nonsinusoidal CPRs existing between pairs of superconducting terminals to realize strong JDE. Supercurrents and JDE efficiency are tunable by magnetic fluxes threading the superconducting loops and by gate electrodes that control the number and transmission of ABSs. One of the loops is further controlled by gating an additional JJ with large critical current, allowing for single-loop operation of the device. Overall, we reach peak JDE efficiency of ±34%. We provide an in-depth explanation of the JDE in our system by means of a simple circuit model, which maps our device to the combination of two SQUIDs, or a bi-SQUID. Simulations are performed both in an idealized case with minimal assumptions and in an extended version that accurately captures the experimental results.

The geometry we engineer can be conceptualized as an interferometer^29^ with one of the two arms constituted by a SQUID. This enables flux tunability over the CPR of the arm and its harmonic content. Consequently, the interferometer exhibits a tunable arm imbalance, leading to the JDE, whose efficiency is further controlled by the second flux degree of freedom. The double flux tunability is a key feature of our platform, as it provides the two sources of symmetry breaking required to implement a superconducting diode: spatial symmetry (here, between two supercurrent paths of the interferometer) and time-reversal symmetry. Local flux bias allows wide and fast tuning of the JDE in both amplitude and sign, including a vanishing diode efficiency in extended regions of the phase space—that is, the JDE can be suppressed without fine-tuning of parameters. Moreover, gating of the hybrid JJs enables electrostatic routing of the supercurrent path and modulation of the flux dependence of the JDE.

In light of our results, multiterminal JJs in superconductor–semiconductor hybrid systems offer advantages that are pivotal for realizing nonreciprocal transport phenomena. The nonsinusoidal, flux-tunable CPR and the ability to break spatial and time-reversal symmetries solely through flux biasing enable the natural attainment of large and controllable diode efficiencies without the need for sizable magnetic fields. Future work could expand the study of multiterminal devices to realize nonreciprocal transport in the linear regime,^47,48^ presenting opportunities for innovative applications.

## Results and Discussion

### Flux-Tunable Multiterminal Josephson Junction

**Figure 1:**
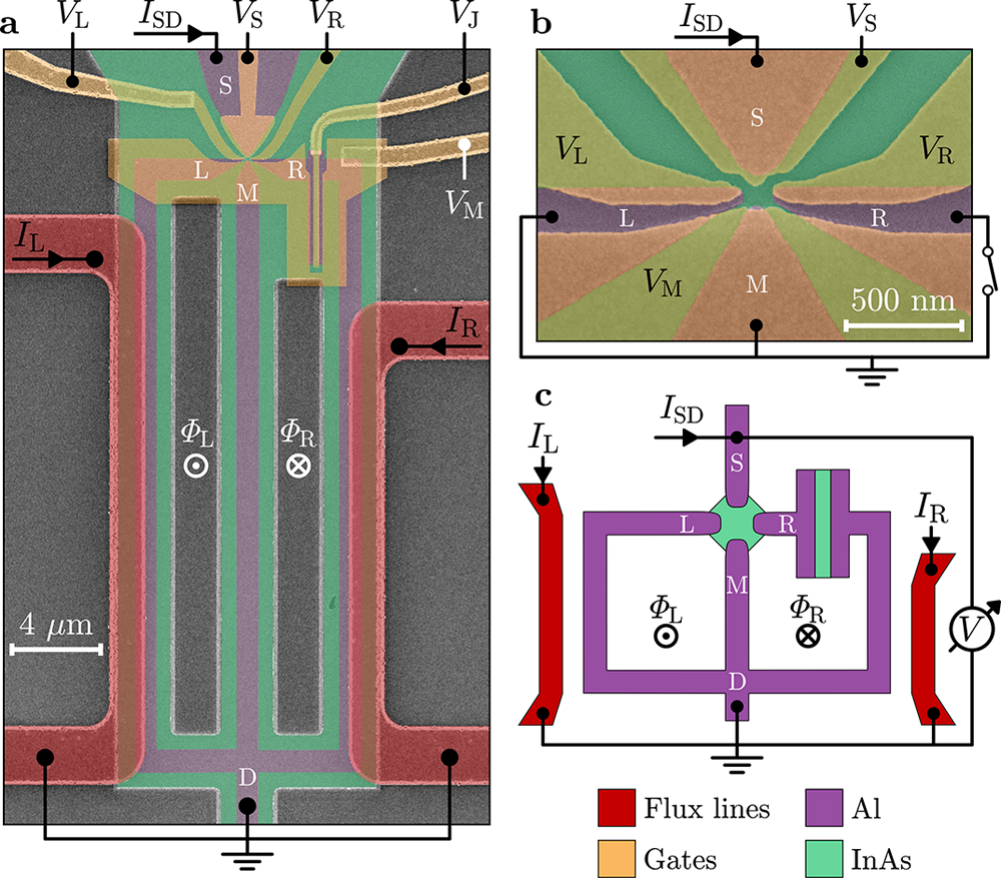
Device under study and measurement setup. (a) False-colored scanning electron micrograph of a device identical to that under study. Exposed III–V semiconductor is represented in green, Al in purple, gate electrodes in yellow, and flux-bias lines in red. Bias current *I*_SD_, flux-line currents *I*_L_ and *I*_R_, magnetic fluxes threading the superconducting loops Φ_L_ and Φ_R_, and gate voltages *V*_S_, *V*_L_, *V*_M_, *V*_R_, and *V*_J_ are labeled. Superconducting terminals S, L, M, R, and the common node D are also indicated. (b) Zoom-in of (a) in the vicinity of the four-terminal Josephson junction. (c) Schematic representation of the device with the measurement setup, using the same color labeling as in (a) and (b). Gate electrodes are not shown.

The device under study, consisting of a multiterminal JJ embedded in double-loop geometry, is displayed in Figure 1. It was realized in an InAs/Al heterostructure,^49,50^ where the epitaxial Al layer was selectively removed to expose the III–V semiconductor below. We defined four superconducting terminals, labeled S, L, M, and R, coupled to a common semiconducting region. Lithographically, the minimum distances between neighboring terminals were 30 nm (for L–M and R–M) and 50 nm (for S–L and S–R), while opposite terminals had separations of 100 nm (L–R) and 120 nm (S–M). All junctions were short with respect to the superconducting coherence length in the InAs 2D electron gas, estimated to be approximately 600 nm (see the Methods section). Terminals L, M, and R were connected to a common node (D) forming two superconducting loops, which enabled independent control over two phase differences,^51,52^ ϕ_L_ – ϕ_M_ ≡ ϕ_L_ and ϕ_R_ – ϕ_M_ ≡ ϕ_R_ (here, ϕ_α_ indicates the superconducting phase of terminal α ∈ {L, M, R} and ϕ_M_ was set to zero by convention). This was achieved by passing currents *I*_L_ and *I*_R_ through two flux-bias lines, patterned on top of a uniform dielectric layer, resulting in external magnetic fluxes Φ_L_ and Φ_R_ threading the left and right loop. Gate electrodes were deposited on the same dielectric layer and energized by voltages *V*_α_ (α ∈ {S, L, M, R}) and *V*_J_, allowing for electrostatic tuning of the electron density in the InAs layer below. While terminals L and M were directly connected to the node D via Al strips, a planar JJ (named switch JJ) was integrated on terminal R. The switch JJ, with a length of 40 nm and a width of 5 μm, was designed to have a critical current much larger than that between any pairs of leads in the 4TJJ, and therefore, the phase difference across the switch JJ can be neglected for the following discussion. Depending on the gate voltage *V*_J_, the switch JJ was employed in two configurations: *V*_J_ = 0 (switch ON), where the JJ was conducting and Φ_R_ could be used to control ϕ_R_, or *V*_J_ = −1.5 V (switch OFF), where the JJ was depleted, the right loop was interrupted, and terminal R was reduced to a floating superconducting island. The other gate voltages were set to *V*_S_ = 0.1 V, *V*_L_ = *V*_R_ = −0.1 V, and *V*_M_ = −0.15 V, unless stated otherwise. The device was measured in a dilution refrigerator with a base temperature of about 10 mK. Current-bias experiments were performed in a four-terminal configuration by driving current *I*_SD_ between S and D and measuring the voltage drop across the device, which allowed for the measurement of the switching current *I*_sw_. Along its path between S and D, the current flowed through the semiconducting region forming the four-terminal JJ, and in particular across the S–L, S–M, and S–R junctions. In our geometry, the superconducting loops were designed to limit their inductance, that could in principle lead to the SDE in the system. The maximum flux variation due to the inductance of a loop, estimated to be approximately 124 pH (see details in Supporting Information, Section 5), for a circulating current on the order of 100 nA, is ∼6 × 10^–3^ Φ_0_. This was observed to be negligible with respect to the flux scales over which the device properties varied. Further details regarding materials, fabrication, and measurement setup are provided in the Methods section. Results on a second device, similar to the one discussed in the Main Text, are presented in the Supporting Information (see Figures S6–S10 in Section 4). Devices studied here were employed in a previous work that investigated hybridization of ABSs in multiterminal JJs.^51^

### Nonreciprocal Supercurrents in the 2D Phase Space

**Figure 2:**
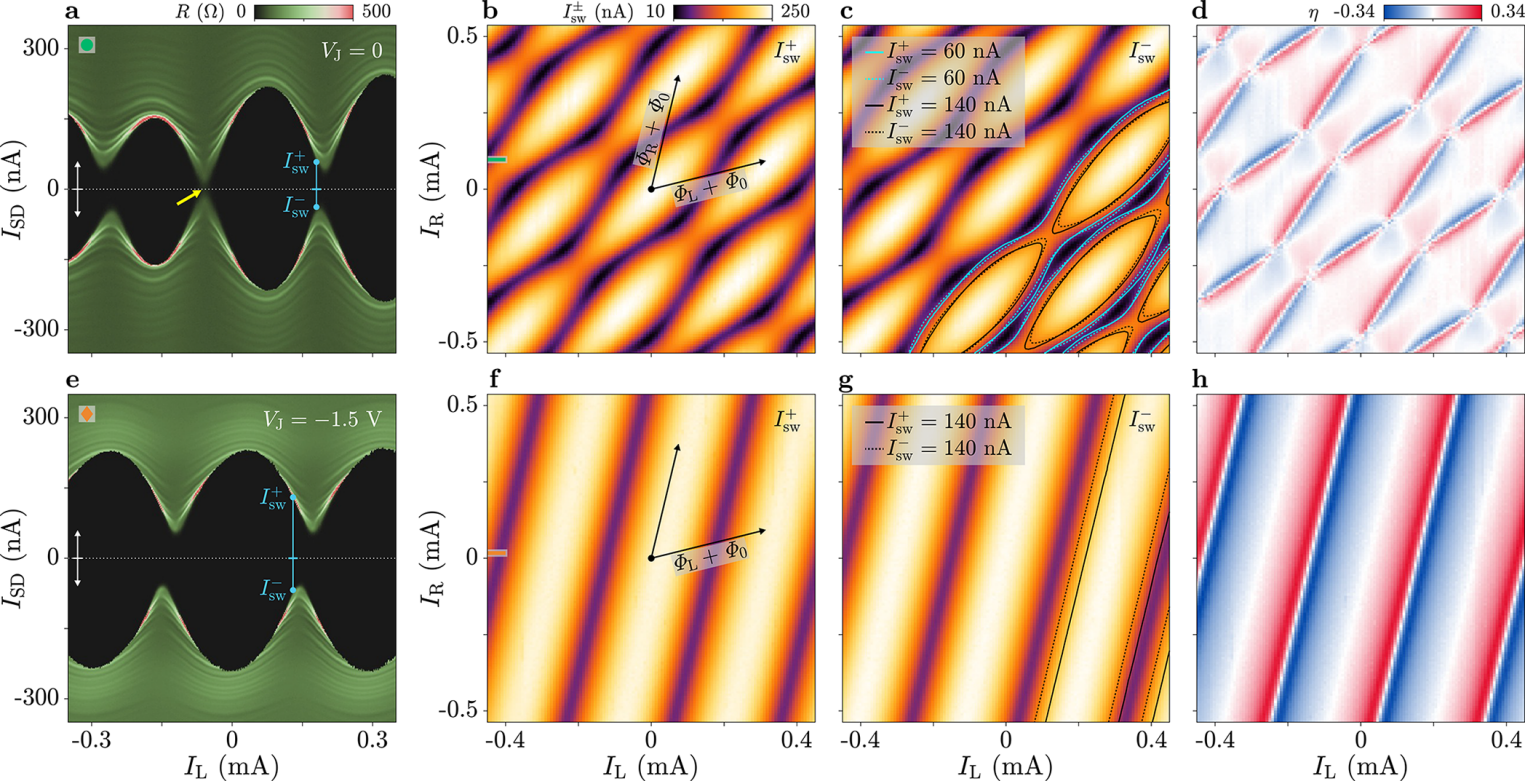
Phase-tunable Josephson diode effect. (a) Differential resistance *R* as a function of left flux-line current *I*_L_ and source–drain bias current *I*_SD_, for fixed right flux-line current *I*_R_ = 0.1 mA. The map is obtained by merging two data sets recorded with *I*_SD_ ramping from 0 to either positive or negative values (see white arrows). Switching current nonreciprocities are highlighted by cyan annotations. A point where the switching current reaches zero is indicated by the yellow arrow. Gate voltages were set to *V*_L_ = *V*_R_ = −0.1 V, *V*_S_ = 0.1 V, *V*_M_ = −0.15 V, and *V*_J_ = 0. (b,c) Switching currents *I*_sw_^+^ and *I*_sw_^–^, measured for positive and negative *I*_SD_ respectively, as functions of *I*_L_ and *I*_R_. Arrows in (b) indicate the directions along which magnetic fluxes threading the left and right superconducting loop, Φ_L_ and Φ_R_, vary independently. Each arrow represents the addition of one superconducting flux quantum Φ_0_ to the corresponding flux. Solid and dotted lines indicate contours of constant *I*_sw_^+^ and *I*_sw_^–^ respectively, for *I*_sw_^±^ = 60 nA (cyan) and *I*_sw_^±^ =140 nA (black). (d) Superconducting diode efficiency η calculated from (b) and (c) using eq 1 (see text), as a function of *I*_L_ and *I*_R_. (e–h) As in (a–d), but measured at *V*_J_ = −1.5 V, which sets the switch JJ to the OFF state and interrupts the right loop. In (e), the right flux-line current is *I*_R_ = 17 μA.

First, we present the differential resistance *R* as a function of the current bias *I*_SD_ and of the left flux-line current *I*_L_ for fixed right flux-line current *I*_R_ = 0.1 mA. Here, *R* was measured with standard lock-in techniques and *I*_SD_ was swept from 0 to positive or negative values to avoid retrapping effects. Figure 2a shows the result for *V*_J_ = 0 (switch ON): the switching current revealed oscillations of varying amplitude as a function of *I*_L_, which, notably, were nonreciprocal at positive and negative *I*_SD_. For instance, at *I*_L_ = 0.18 mA, we measured switching currents *I*_sw_^+^ = 58 nA at *I*_SD_ > 0 and *I*_sw_^–^ = 38 nA at *I*_SD_ < 0 (see cyan annotations), where *I*_sw_^±^ ⩾ 0 by definition. This resulted in a superconducting diode efficiency η, defined as
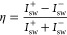
1of approximately 21%. We also note that the switching current vanished in a small range around *I*_L_ ≈ – 60 μA (yellow arrow); namely, the device had finite differential resistance at *I*_SD_ = 0. Similar maps obtained at different settings of *I*_R_ are presented in Figure S1 of the Supporting Information.

To efficiently measure the switching currents *I*_sw_^±^ and the diode efficiency η as functions of both *I*_L_ and *I*_R_, we changed measurement technique and periodically ramped *I*_SD_ from zero to the amplitude *A* = ±260 nA with a repetition rate of 133 Hz, and detected when the voltage drop across the device exceeded a threshold. The time spent in the low-resistance state, averaged over 32 consecutive measurements, was converted to a current, resulting in a rapid measurement of *I*_sw_^+^ or *I*_sw_^–^ (depending on the sign of *A*) displayed in Figure 2b,c, respectively. A limitation of this measurement technique was that values of *I*_sw_ below approximately 10 nA could not be accurately detected due to the finite voltage threshold, which gave a finite reading of about 10 nA for small switching currents and even when the device was resistive for zero bias current. The switching current oscillated periodically in the 2D phase space—where the periodicity axes correspond to the external magnetic fluxes Φ_L_ and Φ_R_ (black arrows in Figure 2b)—forming a pattern characterized by lobe-like features. The finite slope of the Φ_L_ and Φ_R_ axes with respect to *I*_L_ and *I*_R_ was due to the cross-coupling between the left (right) flux-bias line and the right (left) loop, as discussed in Section 5 of the Supporting Information. The oscillations of *I*_sw_ exhibited maxima of approximately 250 nA for Φ_L_ and Φ_R_ equal to integer multiples of the superconducting flux quantum Φ_0_ = *h*/2*e* (with *h* the Planck constant and *e* the elementary charge), and minima at finite phases where the limit of detection was reached, consistent with the vanishing switching current discussed for Figure 2a. We note that the switching currents were nonreciprocal upon reversal of the current bias, while their 2D patterns were symmetric to each other with respect to the origin (*I*_L_ = *I*_R_ = 0, corresponding to Φ_L_ = Φ_R_ = 0). The symmetry was particularly visible in the shape of the lobes, which was inverted as the supercurrent changed sign. In Figure 2c, we plot contours of constant *I*_sw_^+^ (solid lines, obtained from Figure 2b) and *I*_sw_^–^ (dotted lines) for two selected values of the switching current, highlighting the difference depending on the current polarity. Figure 2d shows the superconducting diode efficiency calculated from Figure 2b,c by using eq 1. As expected, η reflected the 2D periodic pattern in the phase space of the switching currents and was widely tunable as a function of *I*_L_ and *I*_R_. We observed a fully ambipolar character and large values up to η ≈ ±21% where *I*_sw_^±^ had a large gradient in the phase space, while the efficiency vanished in extended regions of the phase space without the need for fine-tuning *I*_L_ and *I*_R_.

### Nonreciprocal Supercurrents in Single-Loop Configuration

Next, in Figure 2e–h we present the measurements corresponding to those discussed in Figures 2a–d but with the switch junction in the OFF state (*V*_J_ = −1.5 V). From the differential resistance as a function of *I*_SD_ and *I*_L_ (Figure 2e, here for *I*_R_ = 17 μA), we found periodic oscillations of the switching current, with *I*_sw_^+^ and *I*_sw_^–^ exhibiting a phase shift from each other and opposite skewness (in the forward direction for *I*_sw_^+^, backward for *I*_sw_^–^). Consequently, the switching currents were again nonreciprocal depending on *I*_SD_, which indicates a large JDE; at *I*_L_ = 0.13 mA, for example, *I*_sw_^+^ = 130 nA and *I*_sw_^–^ = 68 nA (see cyan annotations), yielding η ≈ 31%. We note that, in this configuration, the switching current did not vanish for any value of *I*_L_, with minimal values of approximately 50 nA, unlike the case with the switch ON. Measurements of *I*_sw_^+^ and *I*_sw_^–^ as functions of both *I*_L_ and *I*_R_ are shown in Figure 2f,g. The 2D pattern observed for *V*_J_ = 0 was no longer present: as expected, the dependence on the flux Φ_R_ was suppressed when the right superconducting loop was interrupted, and periodicity remained along a single direction. In agreement with Figure 2e, the switching current oscillations were shifted in phase (see solid and dotted lines in panel g, corresponding to constant *I*_sw_^+^ and *I*_sw_^–^ respectively), and their skewness was reversed depending on the sign of the current bias. The superconducting diode efficiency, displayed in Figure 2h for the data of panels f and g, was also characterized by periodic behavior as a function of Φ_L_ and reached maxima of approximately 34%.

### Gate-Tunable Josephson Diode Effect

**Figure 3:**
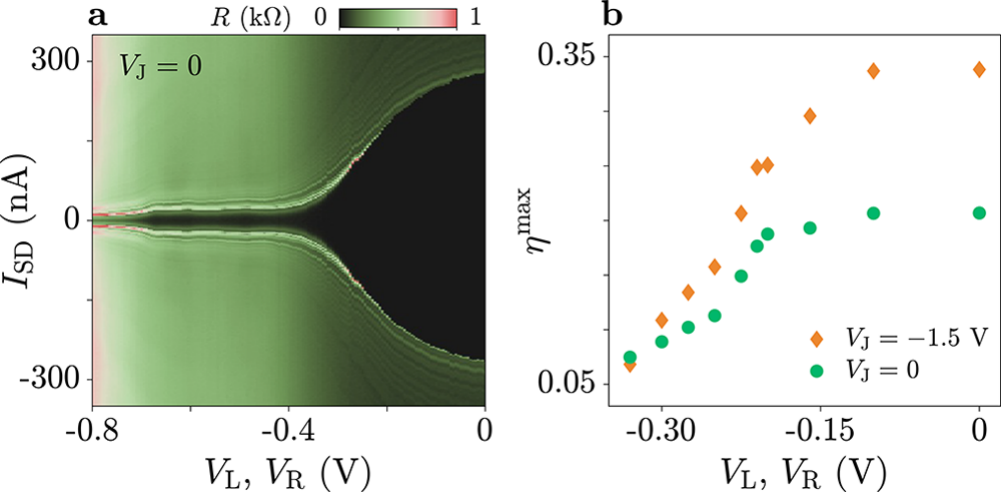
Gate-tuning of the switching current and diode efficiency. (a) Differential resistance *R* as a function of gate voltages *V*_L_ = *V*_R_ and bias current *I*_SD_ (swept from negative to positive values, see white arrow). (b) Maximum Josephson diode efficiency η^max^ as a function of *V*_L_ = *V*_R_. Each point is obtained from data sets similar to Figure 2b,c,f,g (see Supporting Information, Section 3 for more details). Circles refer to the case with *V*_J_ = 0, diamonds to *V*_J_ = −1.5 V.

Electrostatic tunability over the supercurrents and the JDE was enabled by gates controlling the electron density in the semiconducting region of the 4TJJ. In Figure 3a, we show the differential resistance as a function of the current bias while the gate voltages *V*_L_ and *V*_R_ varied simultaneously, for *V*_J_ = 0 and *I*_L_ = *I*_R_ = 0. Here *I*_SD_ was swept from negative to positive values, thus displaying both retrapping and switching currents. The switching current and the retrapping current, respectively 280 and −265 nA at *V*_L_ = *V*_R_ = 0, decreased for more negative voltages, until a finite resistance was measured at *I*_SD_ = 0 for *V*_L_ = *V*_R_ ≈ – 0.35 V. Switching and retrapping currents were very similar for the full gate voltage range, indicating that the transition from resistive to superconducting was governed by phase retrapping, as observed in similar devices.^53^

To investigate the voltage-tunability of the JDE, we measured the switching currents *I*_sw_^±^ as functions of *I*_L_ and *I*_R_ (as in Figure 2b,c,f,g) for varying *V*_L_ and *V*_R_, and, in each configuration, we extracted the peak value of the superconducting diode efficiency, η^max^ (see Supporting Information, Section 3 for the details of the extraction procedure). The result is presented in Figure 3b, where η^max^ is plotted as a function of *V*_L_ = *V*_R_ for the two settings of the switch JJ, *V*_J_ = 0 and *V*_J_ = −1.5 V. In both cases, as *V*_L_ and *V*_R_ increased, we observed an increasing trend of η^max^, which tended to saturate when the gate voltages approached zero. For any gate setting, the diode efficiency was larger at *V*_J_ = −1.5 V than at *V*_J_ = 0, up to 34 and 21% respectively (at *V*_L_ = *V*_R_ = 0).

**Figure 4:**
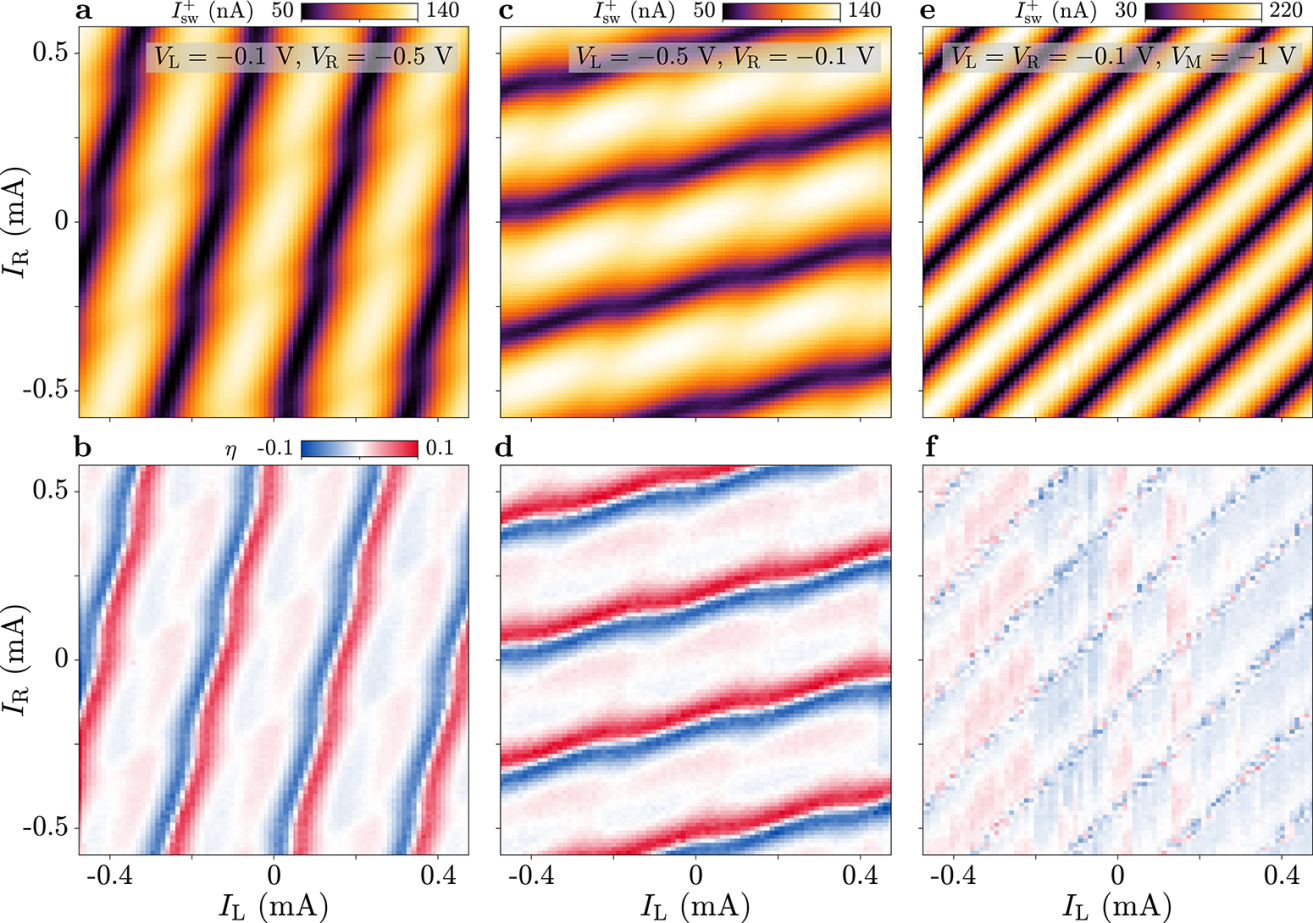
Routing of the supercurrent. (a) Switching current *I*_sw_^+^ measured for positive bias current *I*_SD_, as a function of flux-line currents *I*_L_ and *I*_R_. Measurements are performed with *V*_L_ = −0.1 V and *V*_R_ = −0.5 V. (b) Diode efficiency η for the configuration of (a). (c,d) As in (a,b), but for *V*_L_ = −0.5 V and *V*_R_ = −0.1 V. (e,f) As in (a,b), but for *V*_L_ = −0.1 V, *V*_R_ = −0.1 V, and *V*_M_ = −1 V.

We further characterized the gate dependence of the device by allowing an asymmetric tuning of *V*_L_ and *V*_R_ (at *V*_J_ = 0), as shown in Figure 4a–d for the configurations *V*_L_ = −0.1 V, *V*_R_ = −0.5 V and *V*_L_ = −0.5 V, *V*_R_ = −0.1 V. In each case, the switching current measured as a function of *I*_L_ and *I*_R_ for positive current bias is displayed in the first panel, while the second panel presents the diode efficiency extracted from *I*_sw_^+^ and *I*_sw_^–^. The two configurations revealed complementary behavior: the switching current oscillations and the diode efficiency were almost completely suppressed as a function of Φ_L(R)_ when *V*_L(R)_ was set to a sufficiently negative value, depleting the semiconducting region between terminals S and L (R). This highlights the possibility of routing the supercurrents flowing in our device by gating, which enabled electrostatic control over the phase dependence of the JDE. The results obtained for *V*_R_ = −0.5 V (panels a and b) were reminiscent of those previously observed for *V*_J_ = −1.5 V (Figures 2f–h), where data were independent of Φ_R_.

Finally, we restored the symmetric gate configuration *V*_L_ = *V*_R_ = −0.1 V and studied the effect of depleting the middle gate *V*_M_, set to −1 V (see Figure 4e,f). Here, we observed periodic oscillations of the switching current along a single direction of the phase space, corresponding to the (Φ_L_ – Φ_R_)-axis. The frequency of these oscillations was doubled compared to the case in which *V*_M_ was not depleted (e.g., Figure 2b), consistent with the exclusion of terminal M from the current path. As a consequence, screening currents induced by the flux-bias lines only circulated in the perimeter of the double-loop geometry, leading *I*_L_ and *I*_R_ to control the total flux Φ_L_ – Φ_R_ (note that Φ_L_ and Φ_R_ were defined with opposite signs in Figure 1a,c). Notably, in this symmetric gate configuration where no current flowed into terminal M, the JDE was essentially suppressed (Figure 4f). Results for additional gate settings are shown in the Supporting Information, see Figures S2–S5.

### Minimal-Model Description of the JDE

**Figure 5:**
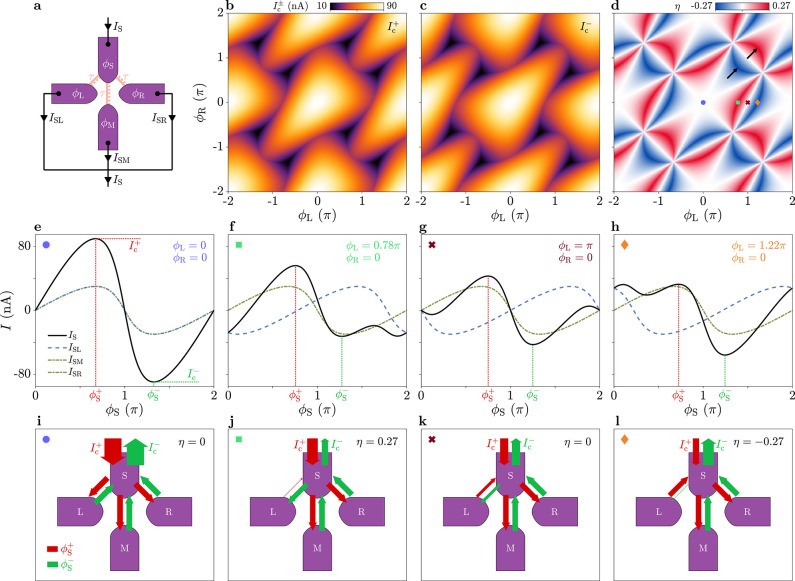
Minimal-model description of the Josephson diode effect. (a) Schematic representation of the four-terminal Josephson junction and circuit parameters. (b,c) Simulated critical currents *I*_c_^+^ and *I*_c_^–^ for positive and negative *I*_SD_, respectively, as functions of the phase differences ϕ_L_ and ϕ_R_. The transmission of the three channels is τ = 0.9. (d) Diode efficiency η derived from (b) and (c) as a function of ϕ_L_ and ϕ_R_. (e–h) Supercurrents in the four leads as functions of phase ϕ_S_. The phases of the other leads are indicated in each panel. The four cases correspond to the colored markers in (d). The value of ϕ_S_ where *I*_S_ has its maximum *I*_c_^+^ (minimum *I*_c_^–^), labeled ϕ_S_^+^ (ϕ_S_^–^), is highlighted by the red (green) dotted line. (i–l) Schematic representation of the supercurrent flow in the phase configurations shown in (e–h). Red and green arrows indicate supercurrents for ϕ_S_^+^ and ϕ_S_^–^, respectively (i.e., *I*_c_^+^ and *I*_c_^–^). The wider the arrow, the larger the supercurrent.

To understand the behavior of our device in more depth and the underlying origin of the JDE, we introduce a simple circuit model that describes the supercurrents of the 4TJJ. We consider the supercurrent that flows from S to D (or from D to S) via the JJs S–L, S–M, and S–R. The remaining JJs L–M, R–M, and L–R are not taken into account as they are shorted by superconducting loops and cannot contribute to the critical current between S and D. That is, the 4TJJ is mapped onto a bi-SQUID as three distinct JJs are connected in parallel. The total current flowing into lead S is thus expressed as
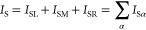
2where *I*_Sα_ is the current flowing from terminal S to terminal α via the corresponding JJ. First, we consider the minimal model of a single numerical parameter, schematically shown in Figure 5a. Each of the three JJs, that are identical to each other, is described by one conductive channel with transmission τ, resulting in the CPR:^54^
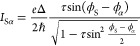
3with Δ the induced superconducting gap (Δ = 180 μeV is used considering Al leads) and *ℏ* = *h*/2π. We assume a high transmission τ = 0.9, for which the CPR of eq 3 has a significantly nonsinusoidal character, as harmonics higher than the first provide a sizable contribution. The independent variables in the model are the three superconducting phases ϕ_L_, ϕ_R_, and ϕ_S_, defined with respect to ϕ_M_ ≡ 0. The first two phases are related to the external magnetic fluxes by ϕ_L(R)_ = 2*πΦ*_L(R)_/Φ_0_ (neglecting the inductance of the loops, see discussion in the Supporting Information, Section 5), whereas ϕ_S_ varies depending on current bias *I*_SD_. The critical currents for the two bias directions are then obtained as
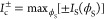
4In Figure 5b,c, we show *I*_c_^±^ computed as functions of ϕ_L_ and ϕ_R_, while the diode efficiency calculated with eq 1 (where *I*_sw_^±^ is substituted by *I*_c_^±^) is displayed in Figure 5d. The critical currents, fulfilling the condition *I*_c_^+^(ϕ_L_,ϕ_R_) = *I*_c_^–^(−ϕ_L_,–ϕ_R_), exhibit patterns that are prominently asymmetric with respect to ϕ_L_ = ϕ_R_ = 0 (modulo 2π), which leads to a strong JDE with η up to approximately 27%. The dependence of η on ϕ_L_ and ϕ_R_ reflects the triangular shapes observed for *I*_c_^±^, with features arranged according to three main orientations in the phase space. The origin of the JDE is investigated by fixing ϕ_L_ and ϕ_R_ and computing the CPR of eq 2 as a function of ϕ_S_, *I*_S_(ϕ_S_), and its components *I*_SL_(ϕ_S_), *I*_SM_(ϕ_S_), and *I*_SR_(ϕ_S_), all obtained from eq 3. For simplicity, we always keep ϕ_R_ = 0 and select four values of ϕ_L_ (colored markers in Figure 5d), where |η| is either zero (ϕ_L_ = 0, π) or maximal (ϕ_L_ = 0.78π, 1.22π). The individual and combined CPRs at these phase-space points are plotted in Figures 5e–h. In each case, we identify the values of ϕ_S_ that maximize the total current *I*_S_ (ϕ_S_^+^, red dotted lines) and its inverse −*I*_S_ (ϕ_S_^–^, green dotted lines), such that *I*_S_(ϕ_S_^±^) = *I*_c_^±^. The currents flowing to and from terminal S are schematically depicted in Figure 5i–l for the same ϕ_L_ and ϕ_R_ values of panels e–h. In the schematics, red (green) arrows show the situation at ϕ_S_^+(−)^, and their width and direction indicate the magnitude and direction of the current. We note that all individual CPRs *I*_Sα_(ϕ_S_) have the same amplitude ≈30 nA and skewness, both given by the transmission τ (identical for the three channels), but notably, *I*_SL_(ϕ_S_) is phase-shifted by ϕ_L_. When ϕ_L_ = 0 (Figure 5e,i), all components are in-phase and *I*_S_(ϕ_S_) = 3*I*_Sα_(ϕ_S_); hence, a standard nonsinusoidal CPR is obtained. Positive and negative critical currents are identical, and all currents are simply reversed between ϕ_S_^+^ and ϕ_S_^–^. In contrast, when *I*_SL_(ϕ_S_) is shifted by ϕ_L_ = 0.78π (Figure 5f,j), the total CPR becomes nonreciprocal for positive and negative currents. The *I*_SL_-component is very small at ϕ_S_^+^ but comparable with *I*_SM,SR_ at ϕ_S_^–^; since in both cases *I*_SL_ has opposite sign with respect to *I*_SM,SR_, this asymmetry leads to *I*_c_^+^ > *I*_c_^–^. A symmetric scenario is recovered for ϕ_L_ = π, when *I*_SL_ is shifted by half a period from the other components. Here, *I*_S_ always has the opposite sign to *I*_SM,SR_, but the same absolute value at ϕ_S_^±^, such that *I*_c_^+^ = *I*_c_^–^ and no JDE is present. Finally, the results obtained for ϕ_L_ = 1.22π (Figure 5h,l), symmetric about ϕ_L_ = π to ϕ_L_ = 0.78π, show the same CPRs discussed for Figure 5f,j upon sign inversion of both current and ϕ_S_, confirming that here the JDE is strong and has opposite directions compared to the previous case.

We note that the JDE has been derived within our minimal model despite the presence of three identical channels, i.e., of the same harmonic content, whereas in a conventional SQUID comprising two JJs the harmonic content must be different between the two JJs.^29^ The multiterminal geometry that we discuss can also be reduced to a conventional SQUID, where two JJs and a phase degree of freedom (for example, the S–M and S–R junctions and ϕ_R_) are replaced by an effective JJ of tunable harmonic content. The effective JJ, together with the third JJ and the remaining phase difference (in the same example, S–L and ϕ_L_), realizes the proposal of ref (29). An important advantage offered by our platform lies in the possibility of phase-tuning the harmonic content of the effective junction, establishing wide and flexible control over the JDE. The nonsinusoidal character of the individual CPRs, which is still a requirement, is obtained in high-transmission hybrid JJs (as those realized in this work), while our geometry eliminates the need for precise control over the transmissions of the single junctions after device fabrication.

### Simulations with the Extended Model

**Figure 6:**
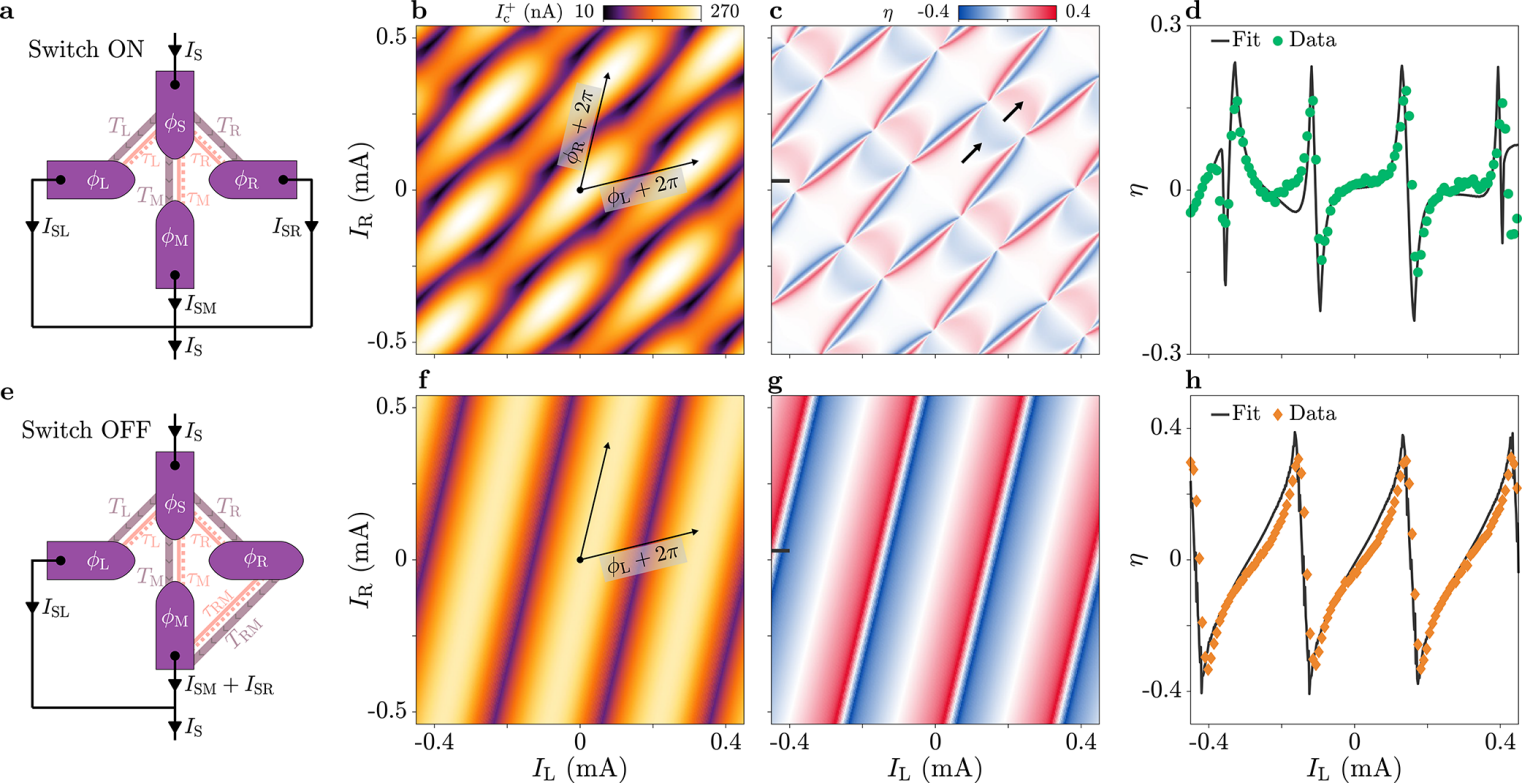
Josephson diode effect in the extended model. (a) Schematic representation of the four-terminal Josephson junction and circuit parameters for the switch-ON configuration (see text for details). (b) Simulated critical current *I*_c_^+^ for positive current bias, as a function of the flux-line currents *I*_L_ and *I*_R_. Currents *I*_L_ and *I*_R_ are calculated from the superconducting phase differences ϕ_L_, ϕ_R_ and the mutual inductance matrix (see details in the Supporting Information, Section 5). Black arrows, whose directions indicate the periodicity axes ϕ_L_ and ϕ_R_, represent the corresponding phase winding by 2π. (c) Diode efficiency η as a function of *I*_L_ and *I*_R_. (d) Comparison of η(*I*_L_) between experimental data (green markers, linecut of Figure 2d at *I*_R_ = 30 μA) and simulation results (black line, linecut of Figure 6c at *I*_R_ = 30 μA). (e) Schematic representation of the four-terminal Josephson junction and circuit parameters for the switch-OFF configuration. (f–h) As in (b–d), but for the case with switch-OFF. In (h), the experimental data (orange markers) are obtained from Figure 2h for *I*_R_ = 30 μA.

After discussing the origin of the JDE by means of a minimal single-parameter model, we extend the latter to better capture the experimental data presented in Figure 2. The extended model, which is schematically depicted in Figure 6a, includes three JJs S–L, S–M, and S–R that contribute to the total current according to eq 2. For each junction we consider two contributions to the current *I*_Sα_: in addition to a high-transparency channel, with transmission τ_α_, a component with conventional sinusoidal CPR,^55^ associated with a large number of low-transmission channels, is included:
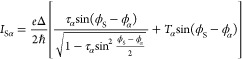
5where *e*Δ*T*_α_/2*ℏ* is the critical current of the sinusoidal component and *T*_α_ the sum of the transmissions of all low-transmission channels. We note that τ_α_ and *T*_α_ may vary depending on junction S−α. This approximation provides a reasonable description of our devices, as spectroscopic measurements revealed a single channel with high transparency accompanied by several channels with significantly lower transparency among neighboring leads.^51^ As in the minimal model, the JJs L−M, R−M, and L−R are shorted by superconducting loops and cannot contribute to the critical current from S to D.

With this extended model, we compute the critical currents using eq 4 for any settings of ϕ_L_ and ϕ_R_. The simulated *I*_c_^+^ and η are shown in Figure 6b,c for parameters τ_L_ = τ_R_ = 0.92, τ_M_ = 0.89, *T*_L_ = 3.5, *T*_M_ = 1.5, and *T*_R_ = 3.6. For a better comparison to the experimental results, the quantities are plotted as functions of flux-bias line currents *I*_L_ and *I*_R_, calculated from the phases ϕ_L_ and ϕ_R_ by applying a linear transformation (see Supporting Information, Section 5 for more details). A direct comparison of the diode efficiency between the experimental data and the simulation results is provided in Figure 6d, where linecuts of η as a function *I*_L_ at *I*_R_ = 30 μA are plotted. Simulations reproduced the measurements displayed in Figure 2 to a good degree. Calculated critical currents were between 10 and 270 nA, with diode efficiencies up to η^max^ ≈ 25% and patterns in the 2D phase space closely resembling the experimental data. By comparing the simulation of η in Figure 6c to the result previously obtained with the minimal model (Figure 5d), we note a reduction of |η| and broadening of the features located near (ϕ_L_, ϕ_R_) = (π, π), modulo 2π (see black arrows in both figures). This effect, also clearly visible in Figure 2d, is mainly related to the sinusoidal components of the CPR of eq 5, where *T*_M_ is substantially smaller than *T*_L,R_ and marginally related to τ_M_, only slightly smaller than τ_L,R_. This is expected in the device under study, as the larger length of the S–M JJ (lithographically of 120 nm) compared to S–L and S–R (50 nm) reduced both the transmission of the highest-transmission mode and the number of channels with low transmission.

The switch-OFF configuration (Figure 2e–h) is investigated by further adjusting the numerical model based on the following considerations. When the right superconducting loop is interrupted, the current flowing from terminal S to R does not have a direct path to D but must flow across the R–M junction. Thus, we must include this junction in the model, with CPR *I*_RM_ also assumed to have the form of eq 5. The current path L–M is still neglected, as it is shorted by the left superconducting loop. In *I*_RM_, the phase difference ϕ_R_ – ϕ_M_ = ϕ_R_ is used instead of ϕ_S_ – ϕ_α_ and parameters τ_RM_ and *T*_RM_ substitute τ_α_ and *T*_α_ (see the schematic of Figure 6e). For these parameters, we choose the values τ_RM_ = 0.97 and *T*_RM_ = 3.2. The other consequence of interrupting the right loop is that ϕ_R_ is not controlled externally with a magnetic flux; hence, it is first calculated imposing the condition *I*_SR_(ϕ_S_ – ϕ_R_) = *I*_RM_(ϕ_R_) (i.e., the current flowing from S to R equals that flowing from R to M), for any value of ϕ_S_. Once ϕ_R_ is determined, *I*_c_^±^(ϕ_L_) is computed using eqs 2, 4, and 5. The result for *I*_c_^+^ is shown in Figure 6f, while the diode efficiency in the 2D phase space is plotted in Figure 6g. A direct comparison between experiment and simulation is displayed in Figure 6h, presenting linecuts of η as a function of *I*_L_ at *I*_R_ = 30 μA obtained from Figures 2h and 6g.

In agreement with Figure 2f,g, the model produces oscillations of *I*_c_ as a function of ϕ_L_ between 50 and 240 nA, with a phase shift when reversing the current direction. This results in a diode efficiency up to 40%, comparable to the measured value of ≈34%, and exhibiting an oscillating behavior depending on ϕ_L_, similar to that in Figure 2h. The higher η obtained in the switch-OFF case is understood by considering the higher asymmetry in the supercurrent distribution obtained in this setting. In fact, the supercurrent flowed directly from S to the common node D only via S–L and S–M, which had largely different transmissions due to the device geometry, while it had to traverse both S–R and R–M to reach D via R. This realized a strongly asymmetric situation, where junctions with different harmonic components led to large diode efficiencies.^29^ When the switch was ON (Figures 2d and 6d) and junction S–R directly connected S to D, the structure of the device became symmetric. In this configuration, the two magnetic fluxes broke both time-reversal symmetry and spatial symmetry in the supercurrent paths, generating the JDE. Similar arguments apply to the case of Figure 4b,d; however, setting gates to negative values to deplete parts of the semiconducting region reduced the maximum switching current, which also resulted in a decrease of the JDE efficiency (see Figure 3). The absence of JDE for the situation of Figure 4f is explained by considering that, with terminal M blocked, the supercurrent flowed in S–L and S–R only, which were almost balanced channels. Furthermore, phase tuning could not break spatial-inversion symmetry with M blocked, effectively resulting in a single superconducting loop between L and R. This situation therefore realized the balanced SQUID device of ref (29), showing no JDE despite the nonsinusoidal CPR of the individual junctions.

We finally discuss the impact on our results of hybridization between spatially overlapping ABSs. Hybridization of ABSs was shown to distort the CPR of individual JJs based on the phase tuning of nearby JJs.^39−41^ In the present devices, hybridization between ABSs was demonstrated by means of local tunneling spectroscopy^51^ between two modes in the L–M and M–R JJs. Since the terminals L, M, and R are shorted by superconducting loops, the ABSs hosted by the L–M and M–R junctions do not contribute to the switching current measured from S to D. Nevertheless, our devices could host more ABSs between S and the other leads that hybridize, giving rise to phase shifts and amplitude modulations in the CPR of individual JJs. In the present experiment, such an effect would be challenging to detect. First, a few high-transmission modes coexist with several low-transmission modes, making the effect of hybridization in the switching current relatively small. Second, our measurements do not detect the CPR of the device and therefore are not sensitive to phase shifts. Instead, the superconducting phases of individual junctions evolve, depending on current bias and flux tuning, until a phase escape event occurs, further complicating the identification of phase changes. The extended model, which does not consider hybridization, reproduces the experimental data to a good degree with realistic JJ parameters and without the need to introduce hybridization. Similar arguments apply to the effect of phase drag^38^ in the InAs region. Phase drag was recently invoked to describe switching current oscillations and nonreciprocal switching currents in a flux-controlled multiterminal JJ with a geometry comparable to that obtained here in the switch-OFF configuration.^37^ As shown by the model of Figure 6e, periodic switching current oscillations are a natural result of the current paths containing a superconducting loop and do not require mesoscopic interactions in the semiconducting region. Similarly, the JDE originates from the nonsinusoidal CPR of the underlying junctions.

## Conclusions

We reported switching current measurements of a 4TJJ in an InAs/Al heterostructure hosting ABSs with large transmission probabilities, resulting in nonsinusoidal CPRs between pairs of terminals. The switching current measured between two contacts showed a strong dependence on the bias current direction, resulting in a JDE. The JDE efficiency could be widely controlled—both in amplitude and sign—by magnetic fluxes, independently tuned via integrated flux-bias lines and gate electrodes, which routed the supercurrent to different transport channels. Other than magnetic fluxes threading the loops, which are electrically generated on-chip, no magnetic field was required. In a first gate setting, where transport through the entire semiconductor region was allowed, the JDE efficiency was periodically modulated by magnetic fluxes, with peak values reaching η ≈ ±21%, including large regions in parameter space with η ≈ 0. When a superconducting arm was interrupted, introducing a larger asymmetry in the supercurrent distribution, a peak efficiency of η ≈ ±34% was reached. The 4TJJ was mapped onto a simple bi-SQUID geometry, with three parallel JJs containing ABSs with large transmission probability. A theoretical model reproduced the experimental observations to a good degree, including switching current and diode efficiency patterns. In our devices, the JDE is a consequence of the nonsinusoidal CPR and the multiterminal geometry, which allows breaking of spatial-inversion symmetry by controlling the magnetic fluxes in the loops. Unlike realizations based on a single loop, an asymmetry between junctions is not required. Our work highlights the potential of phase-tunable multiterminal JJs to engineer JDE with large and widely controllable efficiencies, without the need for exotic materials or external magnetic fields, and underscores the role of these devices as a versatile platform for upcoming applications.

## Methods

### Materials and Fabrication

Devices were realized in a III–V heterostructure grown by molecular beam epitaxy on an InP (001) substrate.^50^ The semiconducting stack (starting from the substrate) consisted of a 1100 nm thick step-graded InAlAs buffer layer, a 6 nm thick In_0.75_Ga_0.25_As layer, an 8 nm thick InAs layer, a 13 nm thick In_0.75_Ga_0.25_As layer, and two monolayers of GaAs. On top, an epitaxial 15 nm thick Al layer was deposited *in situ* without breaking vacuum. A two-dimensional electron gas (2DEG) was confined in the InAs, and its properties were characterized via measurements performed in a Hall bar geometry, which gave an electron peak mobility of 18 000 cm^2^ V^–1^ s^–1^ at an electron sheet density of 8 × 10^11^ cm^–2^. This resulted in an electron mean free path *l*_*e*_ ≳ 260 nm and a superconducting coherence length of the 2DEG proximitized by the Al sheet of , with *v*_F_ the Fermi velocity in the 2DEG and Δ the induced superconducting gap.

In the fabrication process, large mesa structures were first isolated, suppressing parallel conduction between devices and across the middle regions of the superconducting loops. This was done by selectively etching the Al layer with Transene type D, followed by a second chemical etch to a depth of ∼380 nm into the III–V material stack, using a 220:55:3:3 solution of H_2_O:C_6_H_8_O_7_:H_3_PO_4_:H_2_O_2_. Next, features were defined in the Al layer by wet etching with Transene type D at 50 °C for 4 s. The dielectric, uniformly deposited on the entire chip by atomic layer deposition, consisted of a 3 nm thick layer of Al_2_O_3_ and a 15 nm thick layer of HfO_2_. Gate electrodes and flux-bias lines were defined by evaporation and lift-off. In a first step, 5 nm of Ti and 20 nm of Au were deposited to realize the fine features of the gates; in a second step, a stack of Ti/Al/Ti/Au with thicknesses 5, 340, 5, and 100 nm was deposited to connect the mesa structure to the bonding pads and to define the flux-bias lines.

### Measurements Techniques

Experiments were performed in a dilution refrigerator with a base temperature at the mixing chamber of approximately10 mK. The sample was mounted on a QDevil QBoard sample holder system, without employing any light-tight enclosure. Electrical contacts to the devices, except for the flux-bias lines, were provided via a resistive loom with QDevil RF and RC low-pass filters at the mixing chamber stage and RC low-pass filters integrated on the QBoard sample holder. Currents were passed through the flux-bias lines via a superconducting loom with only QDevil RF filters in the mixing chamber stage. Signals were applied to all gates and flux-bias lines via homemade RC filters at room temperature.

In all electrical measurements, a bias current *I*_SD_ was driven between terminal S and node D of the device by applying equal and opposite voltages to S and D via bias resistors, whose resistance was much larger than that of the device under study. The voltage drop across S and D was detected in a four-terminal configuration. Measurements of the differential resistance were performed with lock-in-amplifier techniques by applying a fixed AC current *δI* = 2.5 nA to D in addition to the DC bias *I*_SD_ and detecting the AC voltage *δV* between S and D, thus obtaining the differential resistance *R* ≡ *δV*/*δI*. Measurements of *I*_sw_^±^ were done by periodically ramping *I*_SD_ from 0 to an amplitude *A*, where *A* was positive or negative depending on whether *I*_sw_^+^ or *I*_sw_^–^ was measured; the absolute value of *A* was adjusted depending on the gate configuration to be slightly larger than *I*_sw_^±^. The signal form was a sawtooth wave, applied at a frequency of *f* = 133 Hz using a waveform generator. The voltage drop across S and D was measured with an oscilloscope (averaging 32 measurements), which detected the time interval Δ*t* where the voltage was below a threshold, hence allowing for the calculation of the switching current as *I*_sw_^±^ = |*A*|*f*Δ*t*. The *I*_SD_ values used in this work did not result in significant Joule heating. The dilution fridge line where *I*_SD_ was passed had a total resistance of 5.8 kΩ, mainly due to the RC filters at the mixing chamber level, plus some hundreds of Ohms when the device turned resistive. For *I*_SD_ = 300 nA, the dissipated power was approximately 500 pW, which is negligible. The currents *I*_L_ and *I*_R_ were generated via Yokogawa GS200 units and passed through an RC filter with *R* = 10 kΩ, *C* = 1 μF at the cryostat input. The currents *I*_L_ and *I*_R_ reached the sample via superconducting looms with QDevil RF filters at the mixing chamber level. The resistance of the flux-bias lines, including looms and filters, was approximately 1 Ω and mainly determined by the filters at the mixing chamber. As long as the flux-bias lines remained superconducting, we observed no effects related to Joule heating on the switching current measurements.

The dilution refrigerator was equipped with a superconducting vector magnet, which, despite not being utilized for the experiments, produced a small magnetic field offset. Therefore, small offsets in the flux-bias line currents *I*_L_ and *I*_R_ (up to ∼100 μA) were considered in data sets, in such a manner that the point where *I*_L_ = *I*_R_ = 0 corresponded to a point of the phase space where η = 0 and *I*_sw_^±^ were maximal, as expected when no magnetic fluxes thread the superconducting loops.

## Data Availability

Data presented in this work are available at https://zenodo.org/records/10802361. Further data that support the findings of this study are available from the corresponding author upon request.

## Supporting Information Available

Extended data, including phase-space linecuts at additional values of flux-line currents and results for different gate configurations; procedure to extract maximum diode efficiencies in Figure 3; measurements of a second device, accompanied by simulations performed with the extended model; discussion on the relationships between flux-line currents, external magnetic fluxes, and superconducting phase differences, including an estimation of the inductances in the system (PDF)

## Notes

An initial draft of this work was uploaded to the arXiv preprint server on December 7, 2023, as the following reference: Coraiola, M.; Svetogorov, A. E.; Haxell, D. Z.; Sabonis, D.; Hinderling, M.; ten Kate, S. C.; Cheah, E.; Krizek, F.; Schott, R.; Wegscheider, W.; Cuevas, J. C.; Belzig, W.; Nichele, F.. Flux-Tunable Josephson Diode Effect in a Hybrid Four-Terminal Josephson Junction. arXiv. 2023, 2312.04415. https://arxiv.org/abs/2312.04415 (accessed December 11, 2023).
