# Supplementary material for: Flux-Tunable Josephson Diode Effect in a Hybrid Four-Terminal Josephson Junction
Source: ACS Nano. 2024 Mar 15;18(12):9221–31. doi: 10.1021/acsnano.4c01642 (PMC10976958; doi:10.1021/acsnano.4c01642)
Supplement: Supplementary file 1 — nn4c01642_si_001.pdf [file nn4c01642_si_001.pdf]

Supporting Information: Flux-Tunable Josephson Diode Effect in a Hybrid Four-Terminal Josephson Junction

Marco Coraiola,[†] Aleksandr E. Svetogorov,[‡] Daniel Z. Haxell,[†] Deividas Sabonis,[†] Manuel Hinderling,[†] Sofieke C. ten Kate,[†] Erik Cheah,[¶] Filip Krizek,[†,¶,§] Rüdiger Schott,[¶] Werner Wegscheider,[¶] Juan Carlos Cuevas,[‡,∥] Wolfgang Belzig,[‡] and Fabrizio Nichele[*,†]

†*IBM Research Europe—Zurich, 8803 Rüschlikon, Switzerland*

‡*Fachbereich Physik, Universität Konstanz, D-78457 Konstanz, Germany*

¶*Laboratory for Solid State Physics, ETH Zürich, 8093 Zürich, Switzerland*

§*Institute of Physics, Czech Academy of Sciences, 162 00 Prague, Czech Republic*

∥*Departamento de Física Teórica de la Materia Condensada and Condensed Matter Physics Center (IFIMAC), Universidad Autónoma de Madrid, E-28049 Madrid, Spain*

E-mail: fni@zurich.ibm.com

Contents

1 **Additional Phase-Space Linecuts** **2**

2 **Results for Different V_{L}, V_{R}** **3**

3 **Extracting the Diode Efficiency as a Function of V_{L}, V_{R}** **4**

4 **Results for Device 2** **5**

5 **Current-to-Flux Remapping** **7**

References **9**

Figures **10**

1 Additional Phase-Space Linecuts

The switching current measured for positive bias current I_{sw}^{+} in the phase space (*i.e.*, as a function of both flux-line currents I_{L} and I_{R}), shown in Fig. 2b of the Main Text, is plotted again in Fig. S.1a over extended ranges of I_{L} and I_{R}. Here, in addition to the phase-space linecut $I_{\mathrm{R}} = 100$ μA displayed in the Main Text (green marker), we consider the linecuts $I_{\mathrm{R}} = 20$ μA (white marker) and $I_{\mathrm{R}} = -60$ μA (blue marker). Along these directions, we measured the differential resistance R across the device as a function of I_{L} and of the bias current I_{SD} (always swept from 0 to positive or negative values), and show the result in Fig. S.1b–d. The three linecuts revealed oscillations of the switching current as a function of I_{L}, with maximum switching currents of approximately 240 nA. In all measurements, the supercurrent was nonreciprocal at positive and negative I_{SD}, confirming the presence of Josephson diode effect (JDE) with phase-dependent efficiency η, consistent with Fig. 2d of the Main Text.

2 Results for Different $V_{\rm L}$, $V_{\rm R}$

Figures S.2a–c show three phase-space linecuts, indicated by the colored markers in Fig. S.1a, where the gate voltages $V_{\rm L}$ and $V_{\rm R}$ were set to -0.2 V (while $V_{\rm L} = V_{\rm R} = -0.1$ V in Fig. 2 of the Main Text and in Fig. S.1). The other gate voltages were kept to $V_{\rm S} = 0.1$ V, $V_{\rm M} = -0.15$ V and $V_{\rm J} = 0$. The switching current oscillations qualitatively resembled those described for $V_{\rm L} = V_{\rm R} = -0.1$ V (Fig. S.1) but with reduced amplitude, as the maximum switching current was approximately 170 nA in agreement with Fig. 3b of the Main Text. Regions of vanishing supercurrent (see for example the yellow arrow in Fig. S.2a) were more prominent in this gate configuration, consistent with the larger normal-state resistance. The linecut of Fig. S.2b ($I_{\rm R} = 20$ μA) was also measured with the switch junction voltage set to $V_{\rm J} = -1.5$ V (switch OFF, Fig. S.2d), yielding a picture very similar to Fig. 2e of the Main Text. With respect to the case where $V_{\rm L} = V_{\rm R} = -0.1$ V, we confirm a reduction of the maximum supercurrent (up to about 170 nA) and diode efficiency (up to approximately 25%).

The results shown thus far were acquired with symmetric gate voltages $V_{\rm L} = V_{\rm R}$, except in Fig. 4a–d of the Main Text where either gate was strongly depleted. In these configurations, the currents flowing into terminals L and R were relatively symmetric, as supported by our simulations (Fig. 6), where a good fit was found for $\tau_{\rm L} = \tau_{\rm R}$ and $T_{\rm L} \approx T_{\rm R}$. For $V_{\rm L} = V_{\rm R} = -0.3$ V, we observed switching current patterns that were slightly asymmetric along the two periodicity directions, as shown in Fig. S.3a,b (see cyan arrows in panel a). This was likely due to different lever arm of the gates at voltages $V_{\rm L}$ and $V_{\rm R}$, which created an imbalance in the device for intermediate gate voltages. By setting $V_{\rm L} = -0.28$ V and $V_{\rm R} = -0.31$ V, a more balanced situation was restored (see Fig. S.3d,e). This effect was also visible in the superconducting diode efficiencies, shown in Fig. S.3c,f for the two cases: while the maximum $|\eta|$ slightly varied depending on the periodicity axis at $V_{\rm L} = V_{\rm R} = -0.3$ V, it was substantially more symmetric in the two directions at $V_{\rm L} = -0.28$ V, $V_{\rm R} = -0.31$ V (see black arrows in panels c and f, respectively).

Staying in the gate configuration with $V_\mathrm{L} = -0.28$ V, $V_\mathrm{R} = -0.31$ V, we defined two phase-space linecuts (colored markers in Fig. S.3d, corresponding to $I_\mathrm{R} = 20$ μA and $I_\mathrm{R} = -140$ μA) and measured R along these directions as a function of I_L and I_SD. The result is presented in Fig. S.4a,b, showing switching current oscillations with maxima of about 70 nA and minima where the supercurrent vanished. Nonreciprocity was still present in the switching current depending on the sign of I_SD, despite less markedly than for the previous gate configurations (where V_L and V_R were set to less negative values), consistent with diode efficiencies up to approximately 10% reported in Fig. S.3f. Finally, the gate voltage V_J was set to -1.5 V to operate with the switch OFF, and supercurrent oscillations were measured along the linecut at $I_\mathrm{R} = 20$ μA (see Fig. S.4c), also revealing maximum switching currents close to 70 nA.

3 Extracting the Diode Efficiency as a Function of V_L, V_R

In Fig. 3b of the Main Text, we showed the gate-tunability of the JDE in our devices by presenting the dependence of the maximum diode efficiency η^max as a function of the gate voltages $V_\mathrm{L} = V_\mathrm{R}$. In the switch-ON configuration ($V_\mathrm{J} = 0$), each data point was extracted from measurements of I_sw^+ and I_sw^- taken as a function of both I_L and I_R. Figures S.5a–e and S.5f–j display I_sw^+ and η obtained using Eq. 1 of the Main Text for five selected gate configurations ranging between $V_\mathrm{L} = V_\mathrm{R} = -0.3$ V and $V_\mathrm{L} = V_\mathrm{R} = 0$. Since values close to the limit of detection ($\sim$ 10 nA) gave large variability of the extracted η (see for example Fig. S.3c,f in the regions of phase space where $I_\mathrm{sw}^+ \gtrsim 10$ nA), regions where I_sw^+ was lower than a threshold of 20 nA were not considered. We note that the exact choice of the threshold did not significantly alter the result of the extraction. In each gate configuration, η^max was obtained by considering the 99.9th percentile of $|\eta|$ throughout the corresponding map. In the switch-OFF case ($V_\mathrm{J} = -1.5$ V), included in Fig. 3b of the Main Text, the extraction was

simplified for two reasons: first, since only one periodicity axis remained in the phase space, $I_{\mathrm{sw}}^{\pm}$ could be measured along a single direction (*e.g.*, as a function of I_{L} for any fixed I_{R}), thus improving both speed and resolution of the measurement; second, $I_{\mathrm{sw}}^{\pm}$ did not approach the limit of detection very closely, therefore a threshold was not required to select the data. Similar to the switch-ON case, $\eta^{\max}$ was obtained by considering the 99.9th percentile of $|\eta|$ for each gate setting.

4 Results for Device 2

To further study the JDE arising in a four-terminal Josephson junction (4TJJ) embedded in a double-loop geometry, we characterized a second device fabricated on the same chip. The circuit layout of Device 2 and the geometry of the two superconducting loops were lithographically identical to Device 1 (see Fig. 1a,c of the Main Text). The 4TJJ region, displayed in Fig. S.6, featured a different layout of terminal S (significantly wider than for Device 1), whereas terminals L, M and R were designed to be identical between the two devices. The shape of the gates was also varied, in particular for the gate energized by the voltage V_{S}.

Current-bias measurements were performed as described for Device 1. Figure S.7 shows the switching current $I_{\mathrm{sw}}^{\pm}$ and the extracted diode efficiency η as functions of the flux-line currents I_{L} and I_{R} for three configurations of the gate voltages V_{L} and V_{R} (set to a common value): -0.4 V (a–c), -0.5 V (d–f) and -0.6 V (g–i). The other gate voltages were $V_{\mathrm{S}} = 0.15$ V, $V_{\mathrm{M}} = -0.15$ V and $V_{\mathrm{J}} = 0$ (switch ON). Switching current oscillations in the 2D phase space at $V_{\mathrm{L}} = V_{\mathrm{R}} = -0.4$ V qualitatively resembled those observed for Device 1 in Fig. 2b,c of the Main Text. The larger current maxima and minima, occurring despite voltages were applied in a more negative range than in Device 1, are understood by considering the different geometry of terminal S, that is compatible with a higher number of conduction channels in the S–L and S–R junctions, and a stronger screening of the electric field generated by the gates.

Another feature of Device 2, that was particularly visible at $V_{\mathrm{L}} = V_{\mathrm{R}} = -0.5$ V, -0.6 V, was the large asymmetry between the two periodicity directions Φ_{L} and Φ_{R}. This is attributed to a smaller supercurrent flowing from S to L than from S to R, likely due to a combination of a smaller number and transmission of the modes on the left side of the device, even for symmetrically applied gate voltages. We note that the case with $V_{\mathrm{L}} = V_{\mathrm{R}} = -0.6$ V was qualitatively similar to Fig. 4c,d of the Main Text, where only V_{L} was depleted. The asymmetry was reflected in the diode efficiency η: while oscillations of η in the phase space had some resemblance with those of Device 1 (see Fig. 2d of the Main Text), and also reached large maxima up to $|\eta| \approx 28\%$, they showed different amplitude and features depending on which flux was varied. A prominent example is visible in Fig. S.7c, where features of η near the phase-space points $(\Phi_{\mathrm{L}}, \Phi_{\mathrm{R}}) = (0, \Phi_0/2)$ (modulo Φ_0), marked by the orange arrow, have different shape and smaller η than those near $(\Phi_{\mathrm{L}}, \Phi_{\mathrm{R}}) = (\Phi_0/2, 0)$ (purple arrow). Conversely, for $V_{\mathrm{L}} = V_{\mathrm{R}} = -0.5$ V, -0.6 V, η was larger in proximity of $(\Phi_{\mathrm{L}}, \Phi_{\mathrm{R}}) = (0, \Phi_0/2)$, consistent with the earlier suppression of the Φ_{L}-dependence as the gate voltages were lowered.

Switching currents and diode efficiency of Device 2 were also measured in the switch-OFF configuration ($V_{\mathrm{J}} = -1.5$ V), as shown in Fig. S.8 for $V_{\mathrm{L}} = V_{\mathrm{R}} = -0.5$ V. Oscillations of both $I_{\mathrm{sw}}^{\pm}$ and η, suppressed along Φ_{R}, were similar to those reported for Device 1 in Fig. 2f–h of the Main Text. Interestingly, the maximum diode efficiencies of approximately 12% were significantly lower than in the corresponding switch-ON case (Fig. S.7f), where $\eta^{\max} \approx 25\%$. Again, this is attributed to the asymmetric supercurrent distribution in Device 2, which led to small diode efficiencies along the Φ_{L}-axis and required tuning of Φ_{R} to reach larger values in the case of Fig. S.7f . When the switch was OFF, control over Φ_{R} was disabled, hence reducing $\eta^{\max}$.

The results shown for Device 2 were qualitatively captured by our extended theoretical model, with parameters $(\tau_{\mathrm{L}}, \tau_{\mathrm{M}}, \tau_{\mathrm{R}}, T_{\mathrm{L}}, T_{\mathrm{M}}, T_{\mathrm{R}})$ set to $(0.91, 0.87, 0.93, 3.5, 2, 6.5)$ for Fig. S.9a–c (Configuration 1), to $(0.44, 0.65, 0.87, 1, 1, 3.3)$ for Fig. S.9d–f (Configuration 2), and to

$(0, 0.53, 0.67, 0.2, 0.2, 1)$ for Fig. S.9g–i (Configuration 3). Furthermore, we simulated the device in the switch-OFF case using the same parameters as in Configuration 2 and, in addition, $\tau_{\mathrm{RM}} = 0.75$, $T_{\mathrm{RM}} = 4$; the result is displayed in Fig. S.10. In all cases, the phase axes $(\phi_{\mathrm{L}}, \phi_{\mathrm{R}})$ were converted to flux-line-current axes $(I_{\mathrm{L}}, I_{\mathrm{R}})$ using the transformation described in Section 5. We note that the chosen parameters were such that $\tau_{\mathrm{L}} < \tau_{\mathrm{R}}$ and $T_{\mathrm{L}} < T_{\mathrm{R}}$, supporting our interpretation of the asymmetry between Φ_{L} and Φ_{R} as a result of asymmetric supercurrent flow in the device between terminals L and R. In Configuration 3, the data was best described by using $\tau_{\mathrm{L}} = 0$, suggesting that the high-transmission channel between S and L was depleted. The measurements performed on Device 2 and the simulations done within the same model introduced for Device 1 support the generality of the observed phenomena, and in particular corroborate the presence and origin of the JDE in our device.

5 Current-to-Flux Remapping

The bi-SQUID geometry of our devices enables control over two superconducting phase differences (between terminals L and M, and between R and M), tuned by the currents in the two flux-bias lines, I_{L} and I_{R}. These currents generated magnetic fluxes threading the two superconducting loops, Φ_{L} and Φ_{R}, with a cross-coupling leading to an effect of $I_{\mathrm{L(R)}}$ on the flux through the opposite loop, $\Phi_{\mathrm{R(L)}}$. As a consequence, the Φ_{L}- and Φ_{R}-axes had a finite slope with respect to the I_{L}- and I_{R}-axes, as visible in Fig. 2b of the Main Text. We describe the cross-coupling by considering a mutual inductance matrix $\mathbf{M}$ that relates fluxes Φ_{L} and Φ_{R} to flux-line currents I_{L} and I_{R}:

$$\begin{pmatrix} \Phi_{\mathrm{L}} \\ \Phi_{\mathrm{R}} \end{pmatrix} = \mathbf{M} \cdot \begin{pmatrix} I_{\mathrm{L}} \\ I_{\mathrm{R}} \end{pmatrix} = \begin{pmatrix} M_{\mathrm{LL}} & M_{\mathrm{LR}} \\ M_{\mathrm{RL}} & M_{\mathrm{RR}} \end{pmatrix} \cdot \begin{pmatrix} I_{\mathrm{L}} \\ I_{\mathrm{R}} \end{pmatrix}. \tag{1}$$

To quantify $\mathbf{M}$ (for Device 1), we consider the (Φ_{L}, Φ_{R}) and $(I_{\mathrm{L}}, I_{\mathrm{R}})$ coordinates of two points of the phase space [in addition to the origin, (Φ_{L}, Φ_{R}) $= (I_{\mathrm{L}}, I_{\mathrm{R}}) = (0, 0)$], such as $(\Phi_0, 0)$

and $(0, \Phi_0)$, and substitute them in Eq. 1.[1] The resulting 4×4 equation system leads to the mutual inductance matrix:

$$\mathbf{M} = \begin{pmatrix} 6.98 \text{ pH} & -1.40 \text{ pH} \\ -1.73 \text{ pH} & 5.86 \text{ pH} \end{pmatrix} \tag{2}$$

for Device 1, and

$$\mathbf{M} = \begin{pmatrix} 7.03 \text{ pH} & -1.39 \text{ pH} \\ -1.67 \text{ pH} & 5.80 \text{ pH} \end{pmatrix}$$

for Device 2. We note that $\mathbf{M}$ is very similar between the two devices, as the loop and flux-line geometry was lithographically identical. In Fig. S.11, we apply $\mathbf{M}$ from Eq. 2 to perform a basis transformation and plot the data presented in Fig. 2b–d (shown again in Fig. S.11a–c) as a function of the magnetic fluxes Φ_L and Φ_R (see Fig. S.11d–f).

The inverse transformation, obtained by inverting the mutual inductance matrix, was employed to map the phase space $(\phi_\mathrm{L}, \phi_\mathrm{R})$ to the flux-line-current space $(I_\mathrm{L}, I_\mathrm{R})$ for the simulated data of Figs. 6 (of the Main Text), S.9 and S.10. In this transformation, the phase differences were considered to be linearly related to the fluxes, thus $\Phi_{\mathrm{L(R)}} = \Phi_0 \times \phi_{\mathrm{L(R)}}/2\pi$ were substituted in Eq. 1 to obtain a direct relation between phases and currents. This assumption is justified by noting that the inductance of the superconducting loops, comprising geometric and kinetic contributions $L_\mathrm{loop} = L_\mathrm{geom} + L_\mathrm{k}$, is negligible compared to the Josephson inductance L_J existing between any pairs of terminals in the 4TJJ. For each superconducting loop, the geometric inductance is estimated as $L_\mathrm{geom} \approx 24$ pH,[2] while the kinetic inductance is calculated using the expression:[3]

$$L_\mathrm{k} = \frac{l}{w}\frac{h}{2\pi^2}\frac{R_\Box}{\Delta} \approx 100 \text{ pH}, \tag{3}$$

where $l = 57$ μm is the length of the loop perimeter, $w = 1$ μm the width of the Al strip forming the loop, h the Planck's constant, $R_\Box \approx 1.5$ Ω the normal state resistivity of the

heterostructure stack (measured in Hall bar geometry where the Al was not removed) and $\Delta \approx 180$ μeV is the superconducting gap of Al. Geometric and kinetic contributions lead to a total inductance $L_{\mathrm{loop}} \approx 124$ pH. The Josephson inductance between two superconducting terminals (for example, L and M when considering the left loop) is estimated as $L_{\mathrm{J}} = \Phi_0/2\pi I_{\mathrm{c}} \sim 3$ nH, where $I_{\mathrm{c}} \sim 100$ nA gives the order of magnitude of the junction's critical current.

References

(1) Coraiola, M.; Haxell, D. Z.; Sabonis, D.; Weisbrich, H.; Svetogorov, A. E.; Hinderling, M.; ten Kate, S. C.; Cheah, E.; Krizek, F.; Schott, R.; Wegscheider, W.; Cuevas, J. C.; Belzig, W.; Nichele, F. Phase-Engineering the Andreev Band Structure of a Three-Terminal Josephson Junction. *Nat. Commun.* **2023**, *14*, 6784.

(2) Geometric inductance L_{geom} of a loop was calculated analytically using the following expression, valid for a rectangular loop: $L_{\mathrm{geom}} = \frac{\mu_0}{\pi}\Big[-2(W+H) + 2\sqrt{H^2+W^2} - H\ln\left(\frac{H+\sqrt{H^2+W^2}}{W}\right) - W\ln\left(\frac{W+\sqrt{H^2+W^2}}{H}\right) + H\ln\left(\frac{4H}{d}\right) + W\ln\left(\frac{4W}{d}\right)\Big]$. Here, $H = 26$ μm and $W = 4.5$ μm are the dimensions of the loop, $d = 1$ μm is the width of the Al strip forming the loop and μ_0 is the vacuum magnetic permeability.

(3) Annunziata, A. J.; Santavicca, D. F.; Frunzio, L.; Catelani, G.; Rooks, M. J.; Frydman, A.; Prober, D. E. Tunable Superconducting Nanoinductors. *Nanotechnology* **2010**, *21*, 445202.

Figures

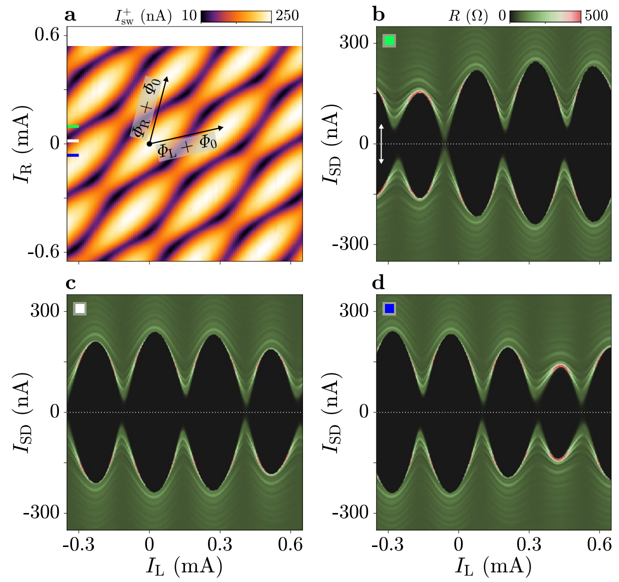

Figure S.1: Phase-space linecuts at $V_L = V_R = -0.1$ V. (a) Switching current I_{sw}^{+}, measured for $I_{SD} > 0$, as a function of flux-line currents I_L and I_R, as in Fig. 2b of the Main Text. Colored markers indicate the position of $I_R = 100$ μA (green), $I_R = 20$ μA (white) and $I_R = -60$ μA (blue). (b–d) Differential resistance R as a function of I_L and I_{SD} for $I_R = 100$ μA (b), $I_R = 20$ μA (c) and $I_R = -60$ μA (d), as indicated in (a). Each map is obtained by merging two datasets measured with I_{SD} ramping from 0 to either positive or negative values [see white arrows in (b)].

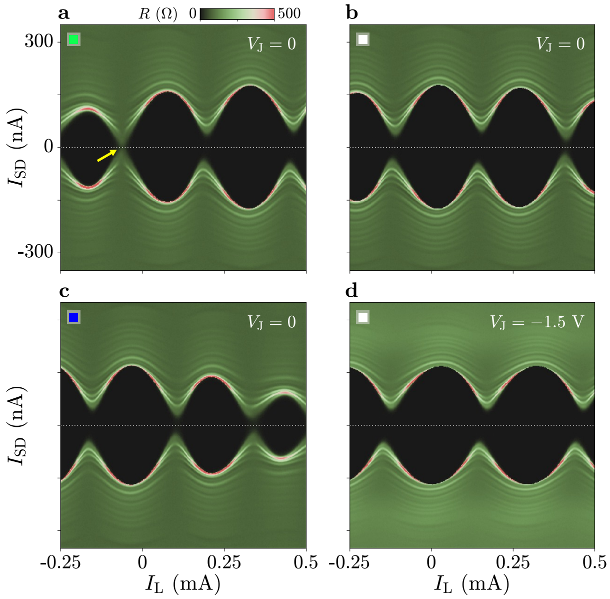

Figure S.2: Phase-space linecuts at $V_L = V_R = -0.2$ V. (a–c) Differential resistance R as a function of I_L and I_{SD}, for $V_J = 0$ (switch ON) and $I_R = 100\ \mu$A (a), $I_R = 20\ \mu$A (b) and $I_R = -60\ \mu$A (c), as indicated in Fig. S.1a. In (a), a point where the switching current reaches zero is indicated by the yellow arrow. (d) As in (b), but for $V_J = -1.5$ V (switch OFF).

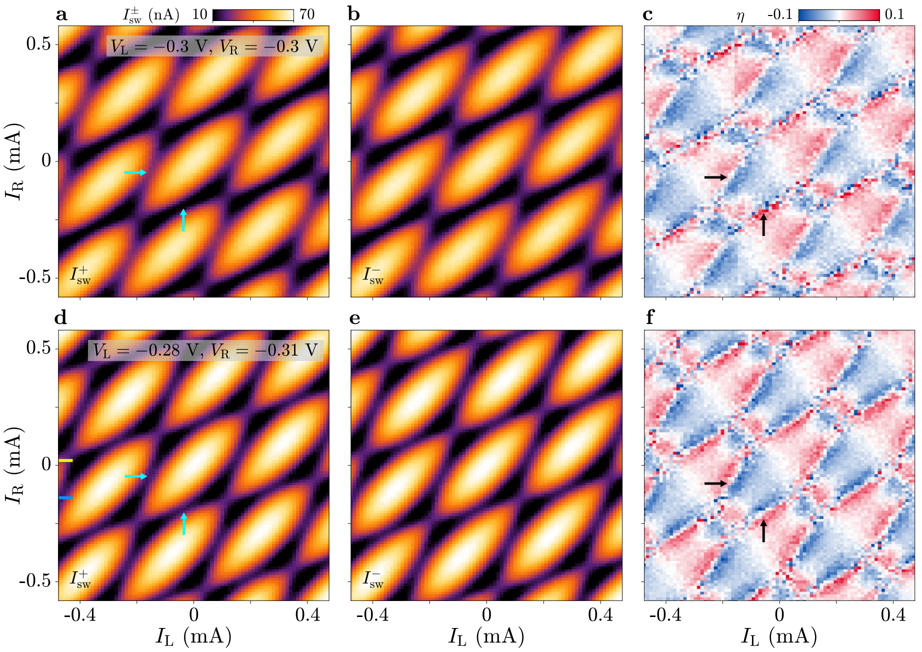

Figure S.3: Comparing symmetric and asymmetric gate configurations. (a, b) Switching currents I_{sw}^{+} and I_{sw}^{-} (respectively) as functions of flux-line currents I_L and I_R for symmetric gate voltages $V_L = V_R = -0.3$ V. The other gate voltages were kept to $V_S = 0.1$ V, $V_M = -0.15$ V and $V_J = 0$. The cyan arrows indicate the switching current minima described in the text. (c) Superconducting diode efficiency η extracted from (a) and (b) as a function of I_L and I_R. Regions indicated by the black arrows are discussed in the text. (d–f) As in (a–c), but for asymmetric gate voltages $V_L = -0.28$ V and $V_R = -0.31$ V. In (d), yellow and blue markers indicate the position of $I_R = 20\ \mu$A and $I_R = -140\ \mu$A, respectively.

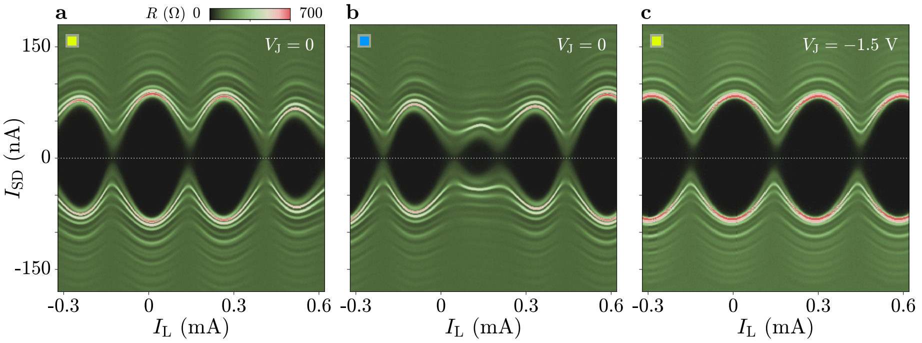

Figure S.4: Phase-space linecuts at $V_L = -0.28$ V and $V_R = -0.31$ V. (a) Differential resistance R as a function of I_L and I_{SD} for $V_J = 0$ and $I_R = 20\ \mu$A (yellow marker in Fig. S.3d). (b) As in (a), but for $I_R = -140\ \mu$A (blue marker in Fig. S.3d). (c) As in (a), but for $V_J = -1.5$ V (switch OFF). Each map is obtained by merging two datasets recorded with I_{SD} ramping from 0 to either positive or negative values.

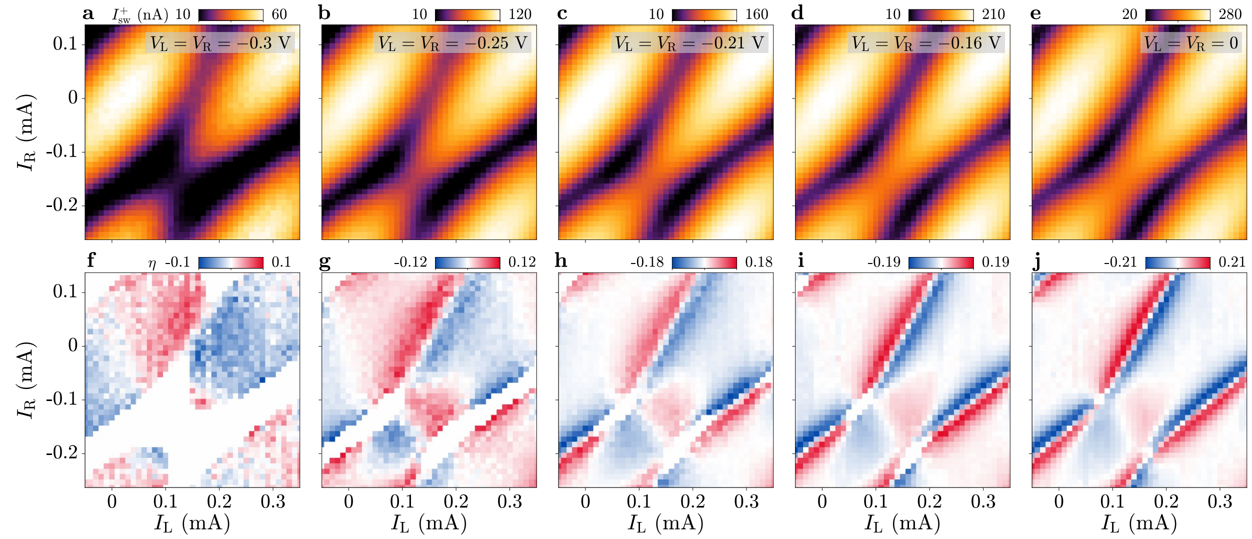

Figure S.5: Results for varying $V_{\rm L}$, $V_{\rm R}$ and extraction of diode efficiencies. (a–e) Switching current $I_{\rm sw}^+$, measured for $I_{\rm SD} > 0$, as a function of flux-line currents $I_{\rm L}$ and $I_{\rm R}$ for five settings of $V_{\rm L} = V_{\rm R}$, indicated on the subfigures. (f–j) Diode efficiency extracted from (a–e) and the corresponding measurements of $I_{\rm sw}^-$ (not shown), respectively, as a function of $I_{\rm L}$ and $I_{\rm R}$. The other gate voltages were kept to $V_{\rm S} = 0.1$ V, $V_{\rm M} = -0.15$ V and $V_{\rm J} = 0$.

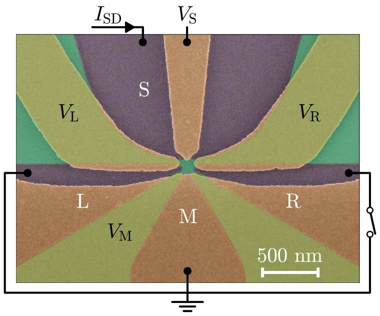

Figure S.6: Device 2: false-colored scanning electron micrograph of the four-terminal Josephson-junction region. Color legend and labeled quantities are as in Fig. 1a,b of the Main Text. Circuit layout and loop geometry of Device 2 are lithographically identical to Device 1 (see Fig. 1a,b).

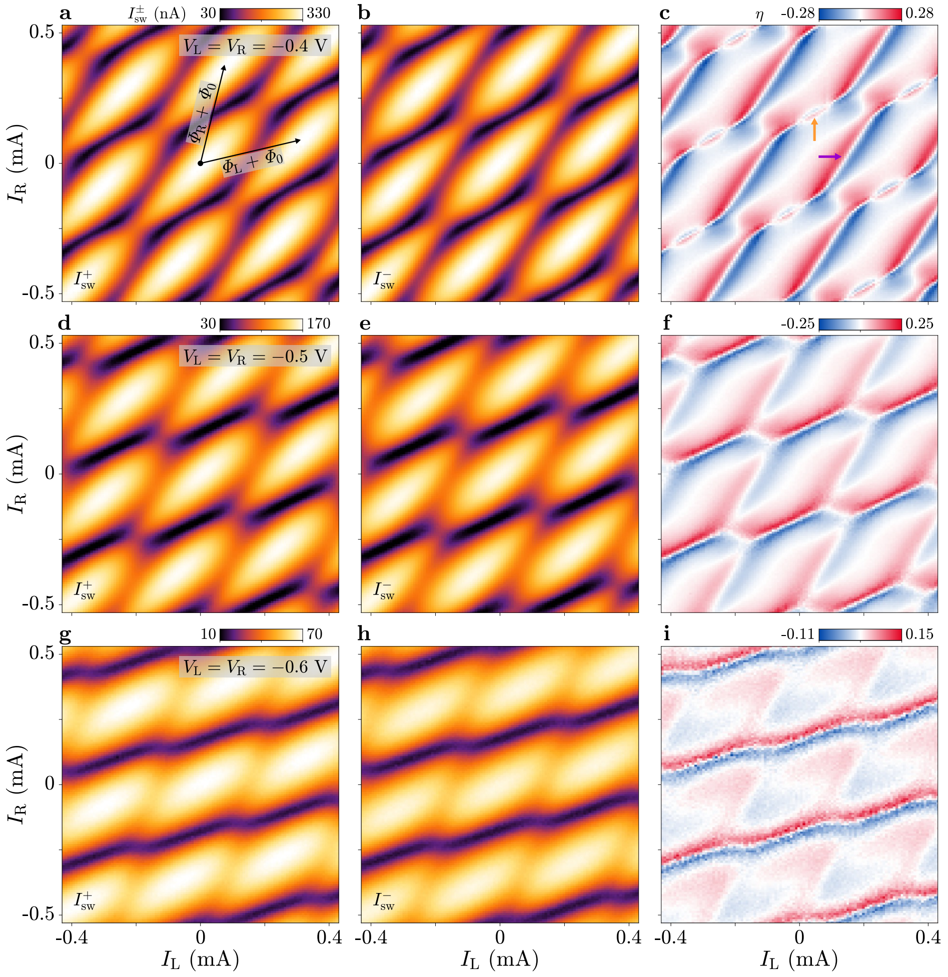

Figure S.7: Phase- and gate-tunable Josephson diode effect in Device 2. (a, b) Switching currents I_{sw}^{+} and I_{sw}^{-}, measured for $I_{\mathrm{SD}} > 0$ and $I_{\mathrm{SD}} < 0$ respectively, as functions of flux-line currents I_{L} and I_{R}, for $V_{\mathrm{L}} = V_{\mathrm{R}} = -0.4$ V. Directions of the black arrows in (a) indicate the periodicity axes, corresponding to the external magnetic fluxes $\varPhi_{\mathrm{L}}$ and $\varPhi_{\mathrm{R}}$ threading the two superconducting loops, while their length (one period) indicates the addition of one superconducting flux quantum $\varPhi_0$ to the corresponding flux. (c) Superconducting diode efficiency η obtained from (a) and (b) as a function of I_{L} and I_{R}. Features indicated by the orange and purple arrows are discussed in the text. (d–f) As in (a–c), but for $V_{\mathrm{L}} = V_{\mathrm{R}} = -0.5$ V. (g–i) As in (a–c), but for $V_{\mathrm{L}} = V_{\mathrm{R}} = -0.6$ V. In all cases, the other gate voltages were set to $V_{\mathrm{S}} = 0.15$ V, $V_{\mathrm{M}} = -0.15$ V and $V_{\mathrm{J}} = 0$.

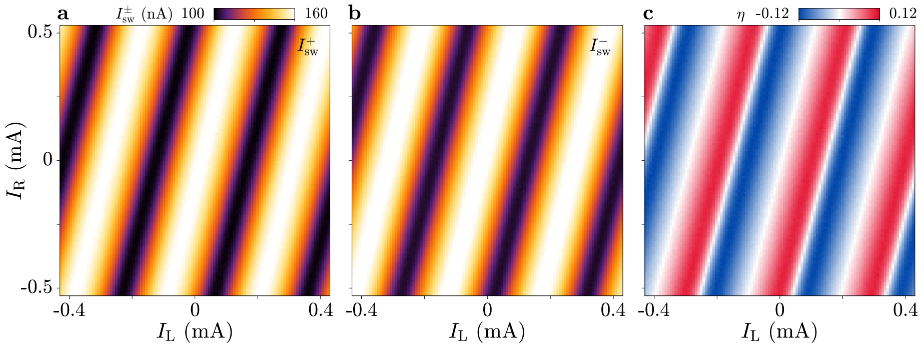

Figure S.8: Josephson diode effect in Device 2 for $V_{\rm J} = -1.5$ V (switch OFF). (a, b) Switching currents $I^{+}_{\rm sw}$ and $I^{-}_{\rm sw}$, measured for $I_{\rm SD} > 0$ and $I_{\rm SD} < 0$ respectively, as functions of flux-line currents $I_{\rm L}$ and $I_{\rm R}$, for $V_{\rm L} = V_{\rm R} = -0.4$ V. Here, $V_{\rm L} = V_{\rm R} = -0.5$ V, $V_{\rm S} = 0.15$ V and $V_{\rm M} = -0.15$ V. (c) Superconducting diode efficiency η obtained from (a) and (b) as a function of $I_{\rm L}$ and $I_{\rm R}$.

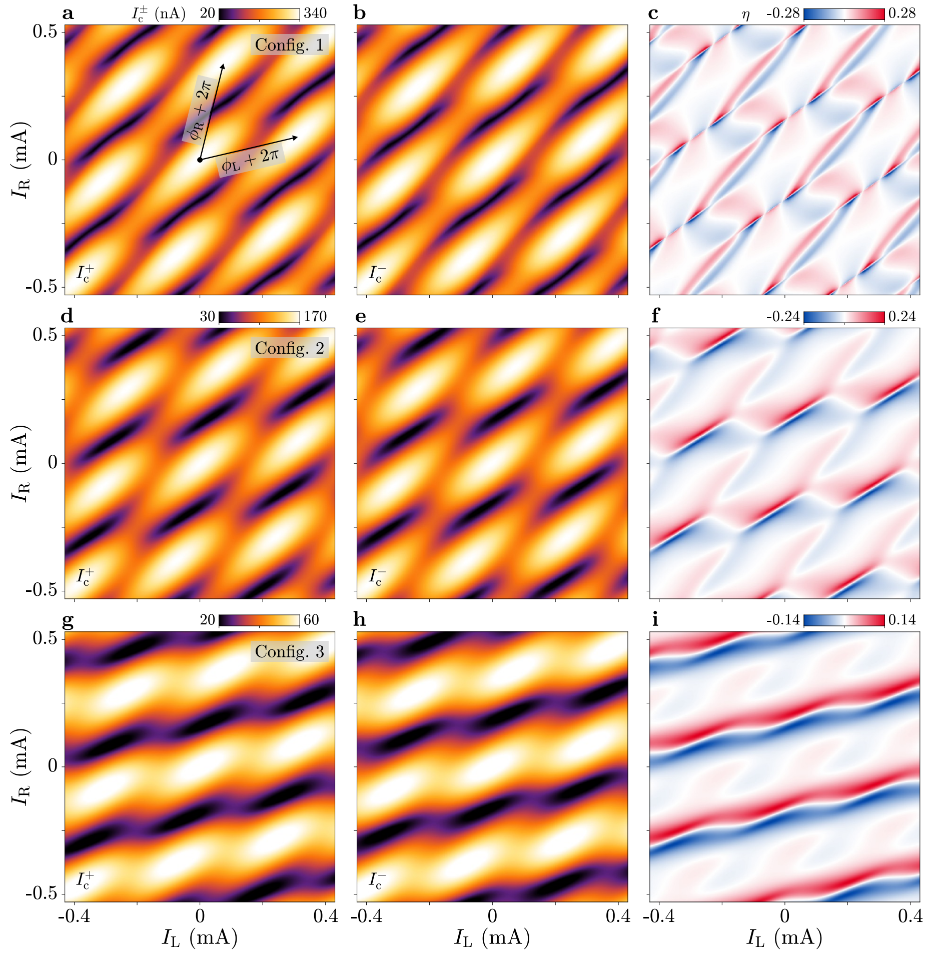

Figure S.9: Simulations with the extended model for Device 2. (a, b) Critical currents I_c^+ and I_c^-, simulated for positive and negative current bias (respectively), as functions of flux-line currents I_L and I_R, using the parameters of Configuration 1 (see text). The model is described in the Main Text. Currents I_L and I_R were obtained from the superconducting phase differences ϕ_L (between terminals S and L) and ϕ_R (between S and R) by applying a linear transformation (see Section 5 for additional details). Phase axes ϕ_L and ϕ_R, that are the periodicity directions, are indicated by the black arrows, whose length represents winding of the corresponding phase by 2π. (c) Diode efficiency η extracted from (a) and (b) as a function of I_L and I_R. (d–f) As in (a–c), but using the parameters of Configuration 2. (g–i) As in (a–c), but using the parameters of Configuration 3.

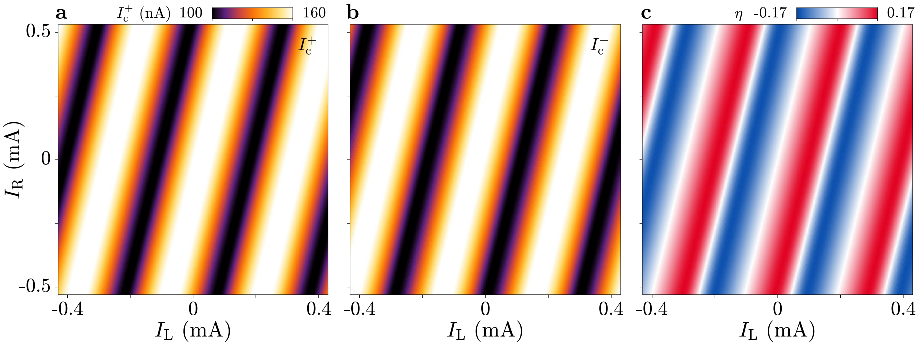

Figure S.10: Simulations with the extended model for Device 2 in the switch-OFF case. (a, b) Critical currents I_c^+ and I_c^-, simulated for positive and negative current bias (respectively), as functions of flux-line currents I_L and I_R, using the parameters of Configuration 2 (see text) and additional parameters $\tau_{RM} = 0.75$ and $T_{RM} = 4$. The model is described in the Main Text. (c) Diode efficiency η extracted from (a) and (b) as a function of I_L and I_R.

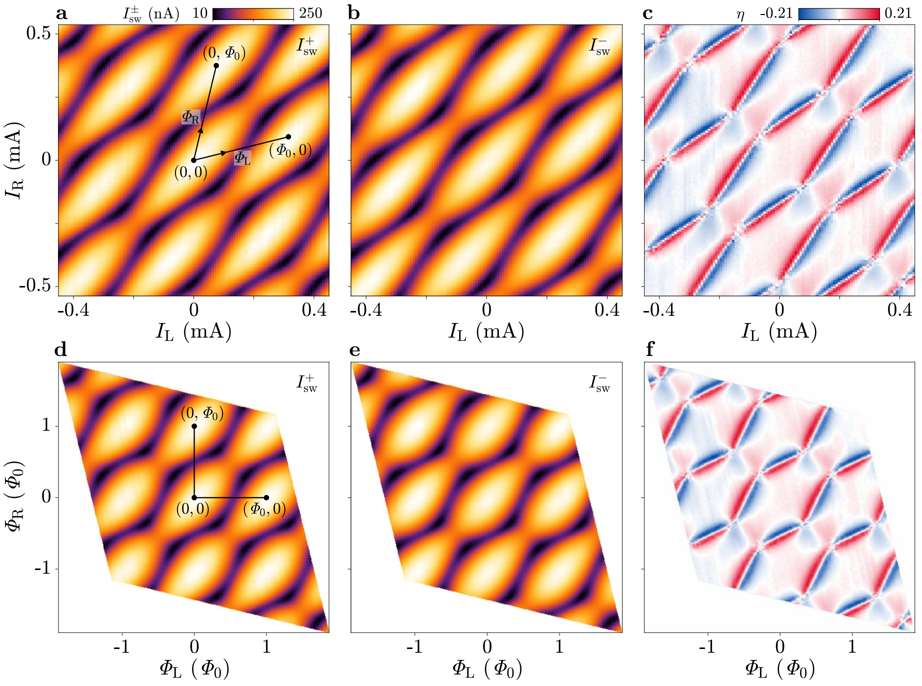

Figure S.11: Linear transformation mapping the flux-line-current space into the flux space. (a–c) Switching currents I_{sw}^+ and I_{sw}^- and diode efficiency η as functions of flux-line currents I_L and I_R, as in Fig. 2b–d of the Main Text. (d–f) Same datasets of (a–c) plotted as functions of the external magnetic fluxes Φ_L and Φ_R, upon applying the linear transformation described in the text.
